# Sustained Self‐Powered Real‐Time Vibration Monitoring Through Integrated Nonlinear Harvesting and Energy‐Aware Wireless Sensing

**DOI:** 10.1002/advs.76967

**Published:** 2026-08-05

**Authors:** Yizhou Li, Ye Zhang, Hao Tang, Yaozi Zheng, Yawei Wang, Chaoyang Zhao, Liwei Dong, Dewen Yu, Junlei Wang, Chunbo Lan, Guobiao Hu

**Affiliations:** ^1^ Thrust of Internet of Things Hong Kong University of Science and Technology (Guangzhou) Guangzhou People's Republic of China; ^2^ School of Civil and Environmental Engineering Nanyang Technological University Singapore Singapore; ^3^ Frontier Institute of Science and Technology Xi'an Jiaotong University Xi'an People's Republic of China; ^4^ School of Mechanical and Power Engineering Zhengzhou University Zhengzhou People's Republic of China; ^5^ School of Renewable Energy Hohai University Nanjing People's Republic of China

**Keywords:** energy‐aware operation, high‐fidelity waveform reconstruction, piezoelectric energy harvesting, quasi‐zero‐stiffness, real‐time monitoring, self‐powered sensing

## Abstract

Continuous high‐frequency data acquisition is critical for advanced structural monitoring and data‐driven systems, yet remains limited by the insufficient energy budget of self‐powered sensing. Existing solutions are largely confined to intermittent operation or low sampling rates, as sustained high‐throughput sensing imposes prohibitive power demands. In this paper, we propose a full‐stack self‐powered sensing framework that overcomes this barrier via a synergistic electromechanical–circuit–sensing co‐design. The system integrates a quasi‐zero‐stiffness (QZS) piezoelectric energy harvester (PEH) for efficient weak‐excitation energy harvesting, a synchronous electric charge extraction (SECE) interface for impedance‐decoupled energy extraction, and an energy‐aware sensing module for efficient high‐frequency operation. This integrated architecture enables sustained real‐time waveform acquisition at a continuous sampling rate of approximately 48 Sa/s, achieving an unprecedented level of continuous high‐frequency sensing compared to existing self‐powered systems and far exceeding the limitations of intermittent operation. The system can operate using energy harvested from a single piezoelectric transducer, while maintaining stable performance over a frequency range of 6–8 Hz at a low excitation level of 0.14 g. By transforming self‐powered sensing from discrete, low‐rate measurements to continuous real‐time monitoring, this work establishes a new paradigm for fully energy‐autonomous sensing and lays the foundation for scalable, infrastructure‐level deployment of intelligent monitoring systems.

## Graphical abstract

A self‐powered vibration sensing platform integrates a quasi‐zero‐stiffness piezoelectric harvester, synchronized electric charge extraction, energy management, and wireless ADC acquisition. Ambient vibration is simultaneously harvested as energy and digitized as information, enabling sustained real‐time waveform monitoring without external power. The system bridges nonlinear energy harvesting and energy‐aware electronics to transform irregular mechanical inputs into continuous sensing.

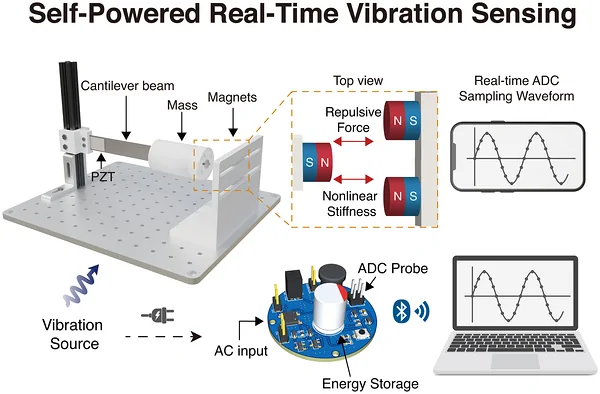

## 1 Introduction

Vibration responses encode rich signatures of structural integrity, making vibration sensing a cornerstone of robust structural health monitoring (SHM) [1]. By analyzing system dynamics, subtle structural degradations, such as localized stiffness loss [2] or micro‐crack propagation [3], can be detected with high sensitivity [4]. Building on this foundation, the integration of SHM with digital twins (DTs) has emerged as a transformative paradigm in the engineering field [5, 6]. Rather than relying on fragmented historical data for retrospective analysis, DTs continuously acquire and assimilate real‐time sensing data to dynamically update internal models [7], enabling accurate representation of the evolving physical system. This seamless physical‐virtual fusion enables a paradigm shift from offline damage detection to real‐time structural state mapping [8].

However, the effectiveness of DT‐enabled SHM critically depends on the availability of continuous, high‐temporal‐resolution data streams [9]. In practice, this requirement introduces a fundamental bottleneck: high‐throughput dynamic data acquisition necessitates sustained and substantial energy input [10]. Conventional sensing infrastructures struggle to meet this demand in a scalable and sustainable manner. Wired systems are limited in scalability and vulnerable to harsh environments [11, 12], while battery‐powered nodes suffer from finite lifespans, maintenance burden, and performance degradation over time [13]. As a result, most deployed systems resort to duty‐cycled or intermittent sensing, inevitably introducing temporal discontinuities and blind gaps that compromise SHM reliability and limit integration with data‐intensive DT frameworks.

To overcome the above limitations, vibration energy harvesting (VEH) has emerged as a promising route toward self‐powered sensing, enabling sensing nodes to operate by converting ambient mechanical energy into electricity [14, 15]. Meanwhile, recent perspectives on battery‐free digital twins further emphasize that self‐powered sensing can transform the physical interface of next‐generation cyber‐physical systems, where energy and information flows are inherently coupled [16]. This paradigm necessitates a tightly coupled co‐design of sensing, communication, and computation under stringent energy constraints, resulting in energy‐aware and inherently intermittent operation. While effective for slowly varying signals, it fundamentally constrains applications requiring continuous, high‐frequency dynamic measurements, where data fidelity and temporal continuity are indispensable.

DTs require heterogeneous data streams acquired at case‐specific temporal resolutions [17]. This intrinsic data diversity, however, introduces uneven energy and sensing burdens, thereby imposing variable challenges for the deployment of self‐powered sensing systems [10]. Despite rapid progress, existing self‐powered systems remain largely limited to monitoring quasi‐static or slowly varying environmental parameters, such as temperature [18, 19], humidity [20, 21, 22], and other low‐frequency signals [23, 24, 25], typically operating in the intermittent, duty‐cycled mode. Although continuous sensing has been demonstrated for slowly varying signals (e.g., temperature) [26, 27], extending these approaches to high‐frequency vibration data monitoring remains fundamentally challenging. Capturing dynamic structural responses, such as displacement and acceleration, requires high sampling rates and continuous data transmission, resulting in an energy burden that is orders of magnitude higher than that of quasi‐static sensing. Due to this intractable energy gap, self‐powered sensing of high‐frequency, data‐intensive dynamic signals remains scarcely explored. Even among the limited pioneering studies that attempt to capture such signals [28, 29, 30, 31, 32, 33, 34, 35, 36, 37, 38, 39], truly continuous operation is fundamentally constrained. To prevent complete power depletion, existing systems are inevitably forced to adopt discrete [28, 29, 30, 31] or duty‐cycled [32, 33, 34] sampling modes, or rely on auxiliary power supply to sustain energy‐intensive sensing circuits [35, 36, 37, 38, 39]. This enforced intermittency severely compromises temporal continuity, leading to incomplete data streams and limiting their applicability in advanced SHM and DT‐related scenarios.

At a fundamental level, the aforementioned limitation stems from the lack of a system‐level integration framework that can jointly optimize energy harvesting, power management, and sensing operations. Existing approaches often treat these components in isolation, without establishing a unified strategy to balance energy harvesting and data demand across domains. Consequently, the intrinsic conflict between energy sustainability and high‐fidelity continuous sensing remains unresolved.

To address this challenge, we propose a synergistic electromechanical sensing co‐design scheme that culminates in a full‐stack and truly self‐powered data‐sensing system specifically engineered to operate in the high‐frequency regime. As envisioned in the conceptual blueprint (Figure 1A), this system establishes seamless real‐time synchronization between the physical infrastructure and its digital counterpart by providing an uninterrupted stream of dynamic data. To realize this grand vision, the physical architecture breaks away from conventional isolated designs, tightly integrating mechanical energy conversion, electrical interface circuitry, and adaptive sensing into a unified system (Figure 1B). On the mechanical front, a quasi‐zero‐stiffness (QZS) energy harvester is engineered to efficiently capture structural vibrations. By superimposing nonlinear magnetic repulsion to counteract the inherent linear positive stiffness, the dynamic stiffness of the system is drastically reduced (Figure 1C). This carefully engineered low stiffness makes the energy harvester exceptionally sensitive to weak vibrations [40, 41]. To overcome the severe energy backflow and source‐to‐load impedance mismatch in standard full‐bridge rectifier circuits, a synchronous electric charge extraction (SECE) interface circuit is seamlessly integrated. Unlike standard full‐bridge rectifiers, the SECE circuit effectively prevents extracted electrical energy from returning to the mechanical domain. It fundamentally decouples power extraction from the load impedance [42], effectively reducing the challenge of impedance matching (Figure 1D). Powered by this robust energy frontend, data acquisition is guided by energy efficiency analysis, precisely matching the high‐frequency ADC sampling rate to the available power budget to balance data fidelity and power consumption. Enabled by the holistic co‐design of these cross‐domain modules, the system establishes parallel energy and information flows as presented in Figure 1E. The energy stream, regulated by the SECE circuit and power management module (PMU), provides a stable supply voltage (*V_DD_*), while a PVDF sensor collects and delivers continuous, real‐time analog signals to the Bluetooth low energy (BLE) programmable system‐on‐chip (PSoC) module. At this nexus, data acquisition is managed through an energy‐aware strategy that continuously aligns sensing activity with instantaneous power availability, thereby enabling sustained high‐frequency data acquisition without compromising system stability.

**FIGURE 1:**
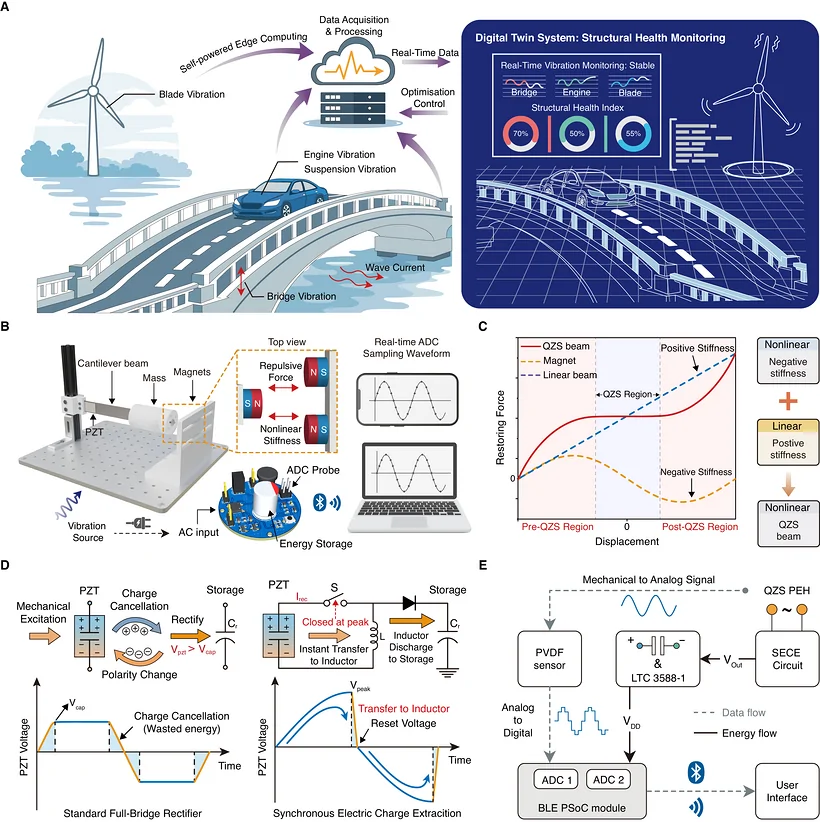
Self‐powered real‐time data acquisition strategy for structural health monitoring. (A) Conceptual framework of the self‐powered sensing system, illustrating the real‐time physical‐virtual synchronization enabled by continuous dynamic data flow. (B) Physical architecture of the fully integrated self‐powered sensing node, demonstrating system‐level integration from the QZS piezoelectric energy harvester, to the synchronous electric charge extraction circuit (SECE), and the wireless sensing node. Dimensional specifications and photographs are available in Figures S1 and S2 and Table S1. (C) Formation of the QZS beam via superposition of linear positive stiffness and nonlinear negative stiffness. (D) Comparison of working principles and charge extraction mechanisms between the synchronous electric charge extraction (SECE) and standard full‐bridge rectifiers. (E) Architecture of the integrated self‐powered sensing workflow, highlighting the synergistic energy management and wireless data transmission.

## 2 Results and Discussion

### 2.1 QZS‐enhanced Energy Harvesting Under Weak Excitation

The QZS energy harvester is achieved by integrating a negative‐stiffness mechanism in parallel with a linear positive‐stiffness element [43]. Figure 1C presents the stiffness profiles of both the negative (magnets) and positive (beam) stiffness components and the resulting QZS behavior after coupling. The structural configuration of the proposed QZS harvester (Figure 2A) consists of a piezoelectric cantilever beam with a cylindrical tip mass at its free end, where a magnet is attached at its distal end. When the tip mass vibrates, the attached magnet interacts with a pair of symmetrically arranged magnets fixed on the support frame, generating nonlinear repulsive forces that counterbalance the beam's positive stiffness and give rise to a desired QZS behavior. The nonlinear magnetic interaction is primarily governed by two key geometric parameters: the horizontal spacing *d* between the moving and fixed magnets, and the vertical distance *h* between the fixed magnet pair (Figure 2A). The resulting flattening of the potential energy wells (Figure 2B,C) indicates a significant reduction in the system's equivalent stiffness near equilibrium. As both *d* and *h* decrease, the QZS region expands. The reduced restoring force relaxes motion constraints, allowing large‐amplitude oscillations under ultra‐low frequency and weak excitations. As *d* further decreases to 13 mm, the negative stiffness provided by the magnets exceeds the positive stiffness provided by the beam near the central position, transforming the potential‐energy landscape from a flattened single well into a double‐well profile with a distinctly convex, non‐flat bottom. This indicates a shift from the monostable QZS regime to a bistable configuration. Based on the potential landscape analysis (Figure 2B,C), we selected *d* = 15 mm and *h* = 12 mm to maximize the QZS effect while preserving a stable monostable equilibrium. This optimal configuration was subsequently used to evaluate the dynamic response and energy‐harvesting performance of the system.

**FIGURE 2:**
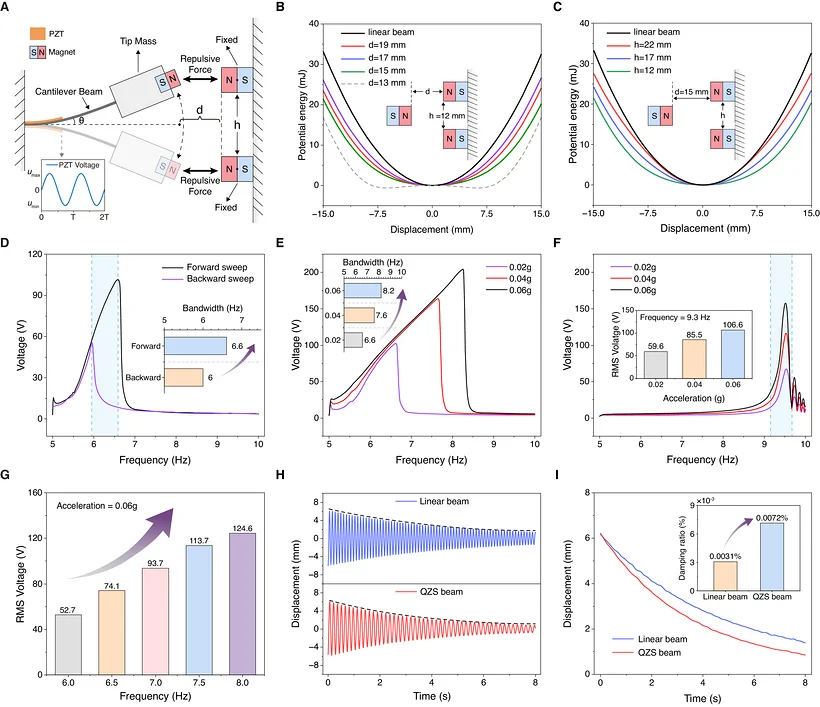
Dynamics characterization of the QZS piezoelectric energy harvester. (A) Schematic of the QZS harvester with magnets arranged to produce repulsive forces. (B,C) Potential‐energy landscapes under varying horizontal magnet distances *d* (B) and vertical magnet spacings *h* (C), illustrating the formation of the QZS region and, at sufficiently small *d*, the transition from a flattened monostable potential to a bistable double‐well potential. The test platform is shown in Figure S3, and further experimental details are provided in Figure S4. (D) Voltage‐frequency response under a chirp excitation with a constant acceleration amplitude of 0.02 g (g = 9.8 m/s^2^), demonstrating the nonlinear hysteresis and the bandwidth asymmetry between forward and backward frequency sweeps. The test platform is shown in Figure S5, and more details are provided in Figure S6. (E) Voltage‐frequency responses under chirp excitations at varying acceleration amplitudes (0.02, 0.04, and 0.06 g), demonstrating nonlinear hardening and bandwidth expansion. See more details in Figure S7. (F) Voltage‐frequency response of a conventional linear energy harvester at varying acceleration amplitudes (0.02, 0.04, and 0.06 g), serving as the narrowband benchmark. See more details in Figures S8–S11. (G) RMS voltage output of the QZS harvester at 0.06 g across different frequencies. Further details are provided in Figures S9 and S10. (H,I) Vibration attenuation performance of the QZS harvester. (H) experimental displacement free‐decay responses and (I) the corresponding displacement‐amplitude envelopes and calculated damping ratios, demonstrating the enhanced mechanical damping of the QZS harvester. The test platform is shown in Figure S16 and more details are provided in Figure S17.

Power output and the working bandwidth are the two primary performance metrics for an energy harvester. Accordingly, the performance of the proposed QZS design is evaluated under varying excitation frequencies and amplitudes (Figure 2D,E). As illustrated in Figure 2D, the forward and backward frequency sweeps reveal clear nonlinear hysteresis. Specifically, the forward sweep exhibits a substantially wider operational bandwidth, extending from 5 to 6.6 Hz, whereas the backward sweep is confined to a narrower range from 5 to 6 Hz. This hysteretic behavior is a defining feature of nonlinear harvesters, arising from the coexistence of multiple stable states within a specific frequency range. During a forward sweep, the system follows the high‐amplitude branch until reaching a critical bifurcation point, where it transitions to the low‐amplitude state. In contrast, during a backward sweep, the system remains on the low‐amplitude branch until transitioning into a low‐amplitude state, thereby forming the observed hysteresis loop. Furthermore, Figure 2E demonstrates that the operational bandwidth of the QZS system progressively expands with increasing excitation amplitude. As the acceleration increases from 0.02 to 0.06 g, the bandwidth widens from 6.6 to 8.2 Hz. This behavior indicates that stronger excitations promote nonlinear hardening, shifting the resonance peak toward higher frequencies and enabling sustained high‐amplitude oscillations over a broader frequency range (Figure 2E). This characteristic benefits practical applications by enhancing the robustness and stability of energy harvesting through the expansion of the high‐energy branch.

In contrast, the traditional piezoelectric cantilever beam exhibits a narrow bandwidth even at elevated excitation amplitudes (Figure 2F). More importantly, the linear beam operates at a significantly higher and fixed resonant frequency of approximately 9.3 Hz. The broadband and low‐threshold harvesting capability of the QZS design is further demonstrated through direct comparison with the traditional linear cantilever beam. As illustrated in Figure 2G, the RMS voltage output under a constant acceleration of 0.06 g exhibits increases from 52.7 to 124.6 V across the frequency range from 6.0 to 8.0 Hz. This performance is further highlighted in comparison with the response of the linear harvester shown in the inset of Figure 2F. The linear harvester delivers a peak RMS voltage of 106.6 V only at a high excitation frequency of 9.3 Hz, whereas the QZS harvester achieves a higher output of 124.6 V at a lower frequency of 8.0 Hz. Furthermore, results of vibration tests (Figures S16 and S17 and Video S1) further corroborate these findings. Under the identical 0.06 g excitation, the QZS harvester exhibits a larger displacement amplitude at 6.75 Hz compared to that of the linear harvester operating at its resonant peak of 9.3 Hz. Notably, over the frequency range of 6.0‐8.0 Hz, where the QZS mechanism operates effectively (Figure 2G), the traditional linear harvester produces negligible output (Figure 2F). This ability to sustain large voltage output at lower frequencies highlights the advantage of the proposed design.

Beyond energy harvesting, the QZS design also exhibits a remarkable vibration‐damping effect (Figure 2H,I). To quantify this behavior, free‐decay tests (Figure 2H) were conducted to compare the mechanical‐to‐electrical energy conversion capabilities of the QZS and linear designs. The QZS design shows a more rapid attenuation than the traditional linear design. This enhanced decay is further confirmed by the voltage envelopes in Figure 2I, where the response of the QZS design drops significantly faster. Quantitatively, the effective damping ratio of the QZS system reaches 0.0072%, exceeding the 0.0031% of the linear counterpart by more than a factor of two (Figure 2I). These results highlight the dual functionality of the QZS harvester in both vibration mitigation and efficient energy harvesting.

### 2.2 SECE Interface for Impedance‐Decoupled Energy Extraction

While the QZS mechanism substantially enhances the mechanical‐to‐electrical conversion efficiency, piezoelectric harvesters inherently suffer from high internal impedance, leading to low current output. This often leads to a significant impedance mismatch when interfacing with a practical electronic load, thereby severely limiting power extraction efficiency and degrading overall system performance. To address the impedance‐mismatch issue, a synchronous electric charge extraction (SECE) circuit is employed as an effective interface strategy. As illustrated in Figures 1D and 3A, under sinusoidal excitation, energy extraction occurs only during the clamping phases of the voltage waveform. The flat‐voltage intervals define the active windows during which the piezoelectric voltage exceeds the voltage across the storage capacitor, turning on the rectifier bridge and enabling efficient charge transfer to the storage capacitor. Outside these intervals, generated charges are largely consumed in charge cancellation rather than being delivered to storage.

**FIGURE 3:**
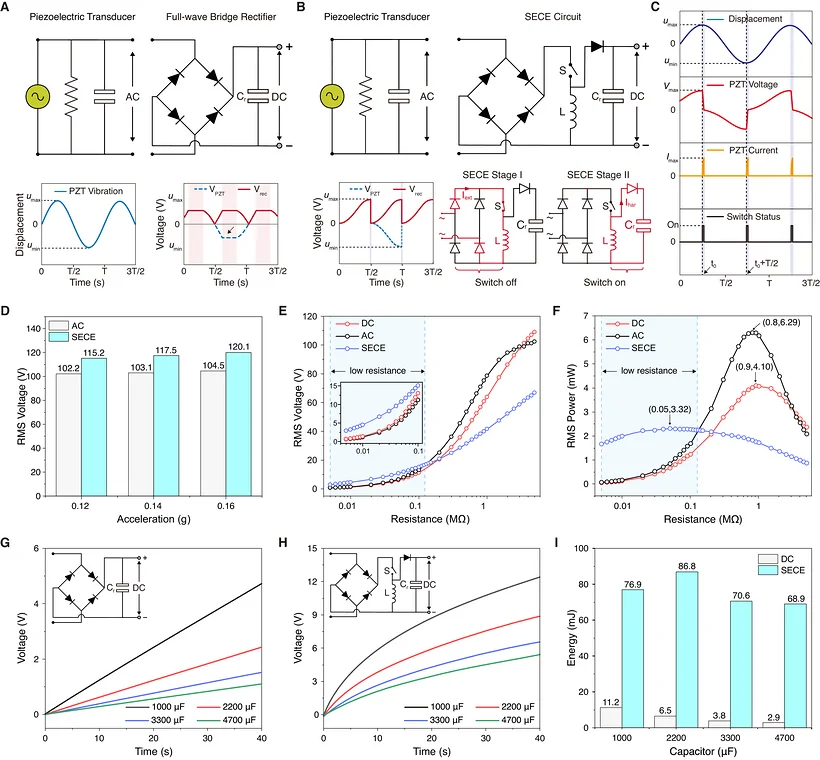
Working mechanism and performance evaluation of the SECE interface circuit. (A) Circuit topology of the standard full‐wave bridge rectifier and corresponding waveforms of displacement and voltage outputs (*V_PZT_* and *V_rec_*). (B) Circuit topology of the SECE interface and its corresponding transient voltage waveforms (*V_PZT_* and *V_rec_*), together with the two‐stage energy extraction process (Stages I and II). See more details in Figure S12. (C) Transient waveforms of SECE operation, showing the synchronization among displacement, voltage, current responses, and the switching actions triggered at displacement extrema. (D) Comparison of RMS output voltages for the standard AC and SECE circuits under excitation accelerations ranging from 0.12 to 0.16 g. See more details in Figures S14 and S15. (E,F) RMS voltage (E) and power (F) as functions of external load resistance for the standard AC, DC, and SECE interfaces. The SECE circuit delivers enhanced voltage (inset in E) and increased power outputs (F) in the low‐impedance regime. (G–I) Capacitor charging curves for the energy harvester using the standard full‐bridge rectifier (G) and the SECE circuit (H), comparison of the accumulated energy after 40 s of charging (I), showing an order‐of‐magnitude energy enhancement by the SECE circuit. Video S2 demonstrates this comparison.

In contrast, the SECE circuit overcomes this limitation by decoupling energy extraction from energy storage. As illustrated in the schematics of Figure 1D, the SECE strategy eliminates the “charge cancellation” phase through an active control of charge transfer. Rather than relying on the voltage to exceed a threshold, the SECE circuit employs a switching interface, consisting of an inductor *L* and an active switch *S*, to initiate energy extraction at moments of maximum potential energy. The circuit topology and key operational stages are detailed in Figure 3B. The transfer of the charge from the piezoelectric transducer to the storage capacitor is controlled by switch *S*, which is triggered near displacement extrema. In stage I (Figure 3B), the switch turns on, establishing an *LC* loop between the piezoelectric transducer and the inductor, allowing the charge in the piezoelectric transducer to be transferred to the inductor. Subsequently, in stage II (Figure 3B), the switch turns off, forcing the inductor to discharge its stored energy and transfer it to the storage capacitor. Notably, after stage I (Figure 3B), the voltage across the piezoelectric transducer is instantaneously reset to almost zero (Figures 1D and 3B), thereby effectively bypassing the charge cancellation process. The dynamic response of this extraction strategy is further illustrated by the time‐domain waveforms in Figure 3C. When the switch is triggered at displacement extrema (*u_max_* and *u_min_*), the piezoelectric voltage *V_PZT_*, which rises continuously to its peak, is abruptly reset to zero. Concurrently, sharp current impulses are observed upon switching, confirming the rapid extraction of accumulated charge.

Additional experiments are conducted to elucidate the superiority of the SECE strategy. Under the same excitation frequency with varying amplitude (0.12‐0.16 g), the piezoelectric transducer integrated with SECE produces a higher RMS voltage (Figure 3D). Notably, even at the lowest excitation level (0.12 g), its output exceeds that of the standard AC interface excited at the highest excitation level (0.16 g). Impedance matching tests are performed to further assess the load‐independent behavior. The SECE circuit sustains a substantially high RMS voltage and power for low electrical loads below 0.1 MΩ (Figure 3E,F), demonstrating strong compatibility with low‐impedance electronics. In contrast, the performance of the standard interface degrades markedly in this regime due to severe impedance mismatch. Although the additional electronic components in the SECE circuit reduce the optimal RMS power compared with the standard interface circuit (from 6.29 to 3.32 mW), it enables the piezoelectric transducer to maintain superior efficiency across a wide range of resistances below 0.1 MΩ, effectively bridging high‐impedance sources and low‐impedance loads. From a practical perspective, capacitor‐charging tests are conducted. Under identical excitations, the SECE circuit consistently outperforms the standard interface across different capacitance values (Figure 3G,H), resulting in a substantial increase in harvested energy (Figure 3I).

### 2.3 Energy‐Aware Sensing for High‐Fidelity Signal Reconstruction

The energy‐efficient wireless sensing node is designed with a streamlined communication architecture. It also incorporates an energy‐aware framework to address the fundamental trade‐off between sampling rate and total power consumption. The wireless data acquisition process is structured into four layers, including the application, host, link, and physical layers to ensure efficient data handling and transmission (Figure 4A). Within the application layer, the ADC subsystem converts analog electrical signals into high‐resolution digital data. It comprises four input channels, where analog signals are sequentially routed through a multiplexer (Input Mux) and a sample‐and‐hold circuit prior to ADC conversion (Figure 4B). Following conversion, the 11‐bit data is encapsulated into a custom payload within the application layer (Figure 4A). The host layer then manages system configuration, allowing key parameters, such as transmission (Tx) power, advertising interval, and channel selection, to be flexibly tuned to balance energy efficiency and communication robustness. The link layer subsequently constructs the protocol data unit (PDU), after which the physical layer appends cyclic redundancy check (CRC) bits and performs Gaussian frequency shift keying (GFSK) encoding to broadcast the packet over the designated RF channels.

**FIGURE 4:**
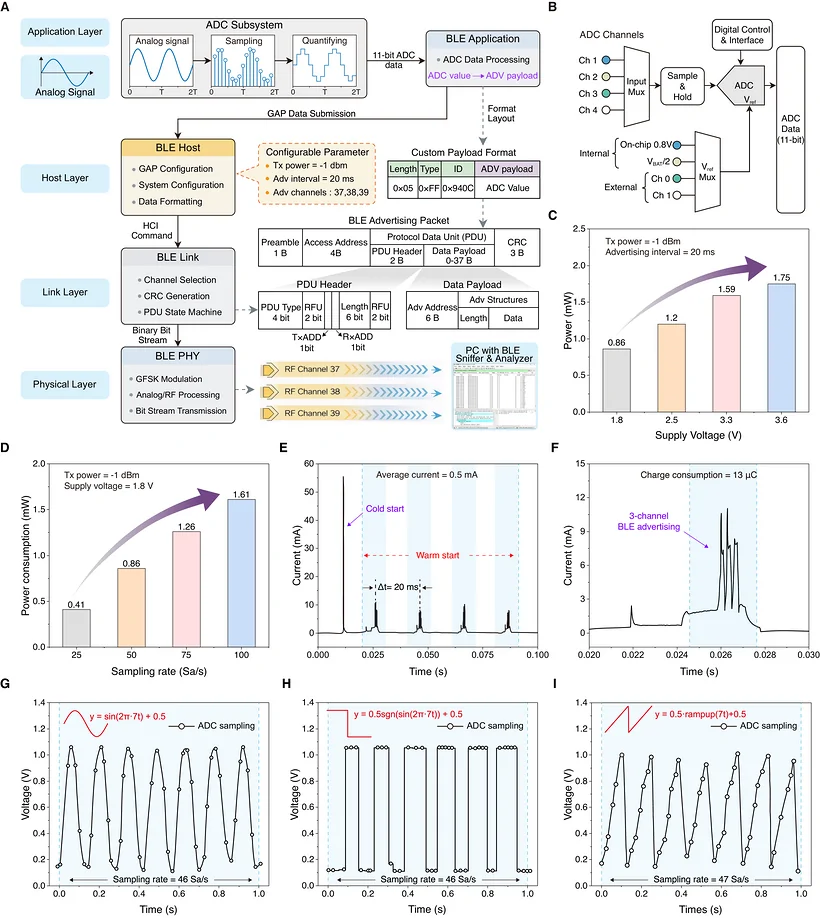
Characterization of the low‐power high‐fidelity wireless sensing node. (A) System architecture of the wireless sensing node, illustrating the data flow from ADC sampling to BLE physical layer transmission. (B) Hardware configuration and block diagram of the 11‐bit ADC module. (C) Power consumption analysis of the sensing node under varying supply voltages. The test platform is shown in Figure S18 and further experimental details are provided in Figure S19. (D) Power consumption analysis of the sensing node under varying sampling rates. See also in Figure S20. (E) Transient current profiles during cold‐start and periodic advertising operation. (F) Charge consumption analysis for a single 3‐channel BLE advertising operation. (G–I) Experimental validation of waveform reconstruction fidelity for (G) sinusoidal, (H) square, and (I) ramp signals.

Power management is central for enabling low‐power sensing and maintaining operational stability. The power consumption of the wireless sensing node was evaluated across a range of regulated supply voltages (Figure 4C). Within the stable operating regime, the node exhibits a minimum power demand of 0.86 mW given a regulated supply voltage of 1.8 V. As the supply voltage increases to 3.6 V, the power consumption rises to 1.75 mW. This increase arises from reduced efficiency of the integrated Low Dropout Regulator (LDO), as a larger input‐to‐output voltage difference results in greater energy loss and lower efficiency. Moreover, an inherent trade‐off exists between the sampling rate and the power consumption of the wireless node. Power consumption was characterized across a range of sampling frequencies, revealing a near‐linear relationship (Figure 4D). Specifically, the power consumption increases from 0.41 mW at a sampling rate of 25 Sa/s to 1.61 mW at 100 Sa/s. Adjusting the sampling rate, therefore, provides a means to reduce energy consumption while maintaining sufficient data resolution for structural health monitoring. However, given a fixed power budget, the maximum sampling rate is fundamentally constrained by the power consumption of the sensing node. Additional acquisition channels, increased computational complexity, or denser wireless communication consume the available power margin, thereby reducing the maximum sampling rate that can be continuously maintained. The current profile under a constant supply voltage of 1.8 V and a sampling rate of 50 Sa/s was recorded to further characterize the dynamic power behavior of the node (Figure 4E). The profile reveals an initial cold‐start phase with a peak current of about 55 mA, followed by periodic warm‐start advertising events. In the steady state, the node wakes every 20 ms to transmit data, sustaining a remarkably low average current of approximately 0.5 mA. Meanwhile, the charge consumption for a single three‐channel BLE advertising event was evaluated (Figure 4F). Each advertising cycle, including hardware wake‐up and BLE data transmission, consumes about 13 µC. Although single‐channel advertising can further reduce power consumption, it may also increase packet loss and degrade data integrity. Therefore, all experiments were conducted using three‐channel advertising over RF channels 37, 38, and 39 to ensure reliable data transmission (Figure 4A).

To validate high‐fidelity signal reconstruction, the wireless node's capability to reproduce diverse waveform morphologies was assessed (Figure 4G–I). As the fundamental frequency of the QZS harvester is centered around 7 Hz, canonical waveforms, including sinusoidal, square, and ramp‐up signals, around this frequency were tested. Despite a relatively low sampling rate of 46–47 Sa/s, the ADC sampling points (hollow circles) exhibit excellent agreement with the ground truth waveforms (red solid lines), demonstrating the node's ability to capture transient features and maintain high signal integrity.

### 2.4 Self‐Sustained Operation for Continuous Real‐Time Monitoring

Fundamentally, the proposed self‐powered sensing strategy is established through the coordinated co‐design of three tightly coupled domains: mechanical energy harvesting using the QZS harvester, efficient power extraction via the SECE interface, and low‐power wireless sensing (Figure 1E). Rather than optimizing these subsystems independently, they are jointly configured to ensure that the usable harvested power under the target vibration conditions can continuously sustain sensing, data processing, and wireless communication. Figure 5A shows the system schematic. A commercial power management unit (PMU, LTC3588‐1) is employed to regulate the harvested energy with minimal loss. Through the SECE interface, which converts the high‐voltage AC output into DC, the extracted energy is stored in a 2.2 mF storage capacitor, and the stored energy is indicated by the voltage across the capacitor, i.e., *V_store_*. For the output configuration, the two voltage selection pins (D0 and D1) are both grounded, setting the PMU to supply a stable voltage of *V_out_* = 1.8 V to the load. This regulated voltage powers the low‐power wireless sensing node, implemented using a BLE PSoC module to minimize power consumption. Serving as the computational core, the node coordinates data acquisition and wireless transmission, while a PVDF sensor captures structural vibrations. To accommodate the limited input range of the on‐board Analog‐to‐Digital Converter (ADC), a resistive voltage divider is employed to scale down the high‐voltage signal.

**FIGURE 5:**
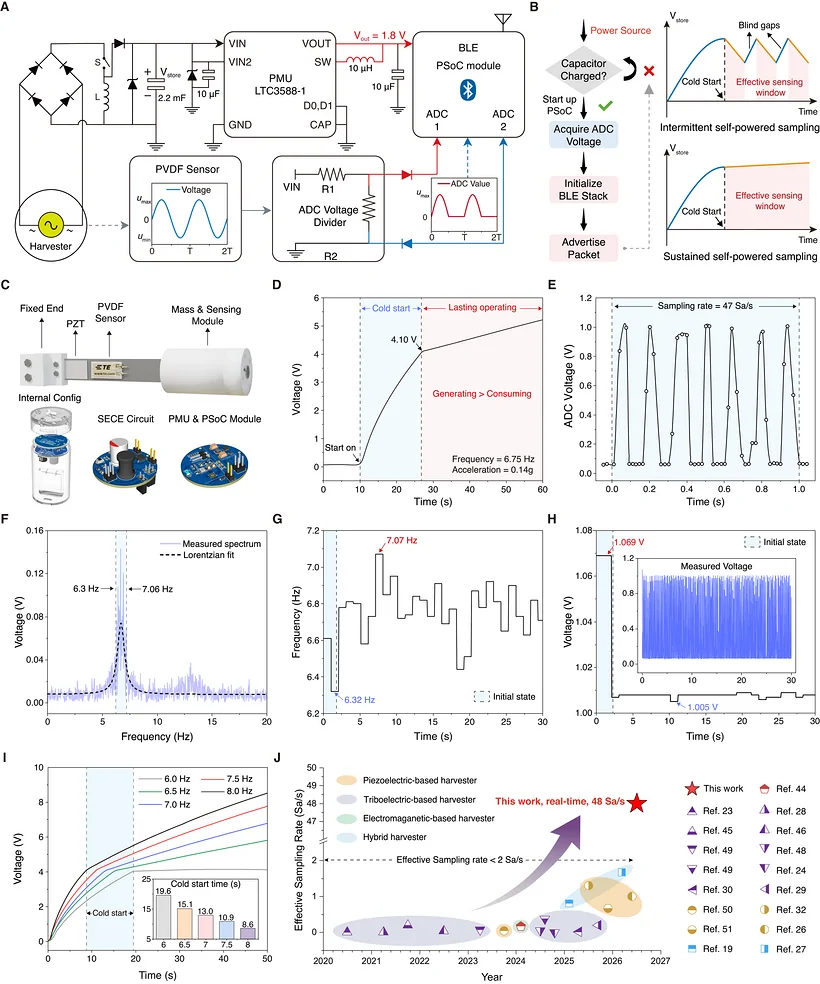
System‐level integration and experimental validation of the real‐time self‐powered sensing system. (A) Schematic diagram of the self‐powered sensing node, integrating a QZS energy harvester, an SECE interface, a power management unit (PMU, LTC3588‐1), a BLE module, a voltage divider, and a PVDF sensor. The detailed experiment implementation is shown in Figures S21 and S22. (B) Energy‐driven sensing workflow, comparing intermittent and sustained self‐powered sampling modes through their capacitor charging–discharging dynamics. (C) Structural assembly and hardware implementation of the sensing node, demonstrating compact integration for real‐time sensing. See also in Figure S13. (D) Storage capacitor voltage profile under an excitation frequency of 6.75 Hz and an acceleration amplitude of 0.14 g, illustrating cold start and sustained operation. (E) Real‐time vibration signal captured by the PVDF sensor and reconstructed wirelessly as ADC voltage at a sampling rate of 47 Sa/s. More details can be seen in Video S3. (F) Corresponding frequency spectrum obtained via Fast Fourier Transform (FFT), identifying the dominant vibration component within 6.3–7.06 Hz. The workflow of the receiving end is shown in Figure S23. (G,H) Real‐time tracking of the (G) vibration frequency and (H) peak ADC voltage at the receiver end, with the inset in (H) showing the full time‐domain waveform. (I) Charging profiles of the storage capacitor under varying excitation frequencies from 6.0 to 8.0 Hz, with the inset showing the corresponding cold‐start duration. (J) Comparison of our developed self‐powered wireless sensing node with existing designs in terms of effective sampling rate and development timeline. See more details in Table S2.

Typically, the PMU operates within a hysteretic window, where *V_out_* is enabled once *V_store_* exceeds the undervoltage lockout (UVLO) threshold of approximately 4.04 V. Conversely, the load is disconnected when *V_store_* drops below 2.87 V to prevent a deep discharge. Governed by this hysteretic window, the self‐powered sensing process follows the workflow illustrated in Figure 5B. The cycle begins with an energy accumulation phase, where the storage capacitor charges until *V_store_* reaches the rising UVLO threshold. Upon triggering, the system wakes up to execute the active tasks, initializing the BLE stack, acquiring ADC data, and broadcasting the advertising packet. If *V_store_* drops below the falling UVLO threshold during operation, the load is immediately disconnected until the capacitor recharges to the rising UVLO threshold, after which the cycle repeats. This cyclic interruption results in an “intermittent self‐powered sampling” mode characterized by “blind gaps” due to an insufficient energy budget (Figure 5B). In contrast, the present system achieves “fully sustained self‐powered sampling” by optimizing the energy budget such that the generated power consistently exceeds consumption. As a result, once *V_store_* surpasses the initial UVLO threshold, the PMU maintains a continuous 1.8 V output without the capacitor voltage dropping below the falling UVLO threshold, thereby eliminating the “blind gaps” observed in conventional intermittent systems (Figure 5B).

The proposed system features a compact physical implementation, with the PVDF sensor directly attached to the beam and the printed circuit board (PCB) integrated into the cylindrical tip mass (Figure 5C). This design eliminates external wiring and effectively reduces the overall footprint. The capability for sustained self‐powered sampling is experimentally validated, and the results are presented in Figure 5D. Under an excitation of 0.14 g at 6.75 Hz, the *V_store_* rises from zero, completing the “cold start” phase in approximately 15.4 s. Notably, after the PMU activates the load at 4.10 V, *V*_store_ continues to rise, offering direct experimental evidence that the system maintains a positive energy balance after node activation. This result confirms that the energy harvested from the monitored vibration is sufficient to continuously sustain ADC acquisition, data processing, and BLE transmission of the corresponding vibration information. With this stable power budget established, the sensing performance of the wireless node is subsequently evaluated. Figure 5E shows a representative segment of the time‐domain vibration waveform, reconstructed from the wirelessly transmitted data packets at a sampling rate of 47 Sa/s. The high quality of the acquired signal allows for precise spectral analysis. As shown in Figure 5F, the measured spectrum aligns perfectly with a Lorentzian fit, accurately identifying the dominant vibration frequency over 6.3‐7.06 Hz. Moreover, the system enables continuous real‐time monitoring. As shown in Figure 5G,H, the vibration frequency and peak voltage amplitude are continuously updated at the receiving terminal, showcasing the system's strong capability for uninterrupted, real‐time sensing. Additional experiments are conducted to evaluate the operational bandwidth for sustained self‐powered sampling. The system maintains robust operation within a frequency range of 6–8 Hz. Notably, higher excitation frequencies enhance energy extraction efficiency, shortening the cold‐start time from 19.6 to 8.6 s (Figure 5I). Compared with existing state‐of‐the‐art self‐powered sensing systems (Figure 5J and Table S2) [19, 23, 24, 25, 27, 28, 29, 30, 32, 44, 45, 46, 47, 48, 49, 50, 51], the proposed system unprecedentedly achieves continuous monitoring capability and increases the sampling rate. Previous studies are constrained by an intractable trade‐off between temporal continuity and high‐sampling data acquisition, limiting them to intermittent operations with effective sampling rates below 2 Sa/s. In contrast, the proposed synergistic co‐design strategy can overcome this limitation. By achieving a sustained sampling rate of 48 Sa/s, this work advances self‐powered sensing from discrete snapshot measurements to continuous real‐time data acquisition. This unprecedented temporal continuity meets the stringent high‐throughput data demands essential for seamlessly bridging the physical‐virtual gap in real‐time battery‐free digital twins. Beyond the demonstrated performance, the potential effects of long‐term cyclic degradation and temperature‐induced QZS drift on the harvested‐power margin and sustained operating capability are further discussed in Note S1.

## 3 Conclusion

In this work, we present a full‐stack, self‐powered sensing framework that fundamentally addresses the long‐standing energy bottleneck in continuous, high‐frequency data acquisition, operating entirely without external power sources. By tightly integrating a quasi‐zero‐stiffness (QZS) piezoelectric energy harvester (PEH), a synchronous electric charge extraction (SECE) interface, and an energy‐aware wireless sensing node, the proposed system resolves the intrinsic trade‐off between temporal continuity and sampling fidelity. Through system‐level co‐design, sustained, real‐time waveform acquisition is experimentally achieved at a continuous sampling rate of approximately 48 Sa/s, marking a substantial advance beyond self‐powered intermittent sensing. Notably, unlike most existing designs that depend on increased device scale or multiple harvesters (Table S2), this system operates using energy from a single piezoelectric transducer. Importantly, its sustained operation is realized without any stringent operating requirements and remains stable over a relatively broad frequency range of 6–8 Hz at a weak excitation level of just 0.14 g, highlighting its strong practical applicability. More broadly, this work represents a paradigm shift in self‐powered sensing, transforming it from discrete measurements to real‐time monitoring. By enabling uninterrupted high‐throughput vibration data acquisition, the proposed system provides a viable physical foundation for establishing real‐time digital twins. It also paves the way toward scalable, autonomous structural health monitoring with persistent, infrastructure‐level deployment.

## 4 Methods

### 4.1 Device Fabrication

The cantilever beam was fabricated from a manganese steel plate with a width of 22 mm and a thickness of 0.8 mm. A cylindrical tip mass was attached to the free end of the beam and fabricated via fused deposition modeling (FDM) using a commercial 3D printer (Bambu Lab X1E) with polylactic acid (PLA). The 3D‐printed structure comprises two segments: one for mechanical connection to the beam and the other for mounting permanent magnets. An N52 NdFeB magnet was fixed at the top of the cylindrical tip mass, while two additional NdFeB magnets were mounted within a 3D‐printed supporting frame. These magnets are arranged to generate repulsive interactions with the magnet on the cylindrical mass, forming the quasi‐zero‐stiffness configuration. A PZT patch was bonded onto the surface of the cantilever beam using epoxy resin and cured for 24 h to ensure stable mechanical coupling. The electrical subsystem, including the SECE interface, energy management module, and data‐acquisition circuits, was implemented on a custom‐designed printed circuit board (PCB). The PCB board was embedded within the 3D‐printed structure, serving both as part of the cylindrical mass and as the functional electronics unit, enabling compact electromechanical integration. The entire experimental setup was constructed using an aluminum‐profile frame to ensure structural stability and precise alignment during testing.

### 4.2 Integrated Circuit Implementation

The integrated circuit consists of three functional modules: an energy extraction interface, an energy management module, and a data acquisition and wireless transmission module. The circuit was implemented on a custom‐designed PCB fabricated by Shenzhen JLC Technology Group Co., Ltd.

The energy extraction interface adopts a synchronous electric charge extraction (SECE) topology, incorporating a 10 mH inductor for charge transfer and a 1000 µF capacitor for energy storage. The energy management module is realized using a commercial power management unit (LTC3588‐1), which features an under‐voltage lockout (UVLO) function with a hysteretic window of approximately 1 V. This configuration enables rapid cold start once sufficient energy is accumulated in the storage capacitor and then maintains stable operation within the hysteresis window, even under fluctuating input conditions.

### 4.3 Characterization and Measurement

A series of experiments was conducted under controlled conditions to comprehensively evaluate the performance of the proposed system. The force–displacement relationship of the cantilever beam was characterized using a digital force gauge (HP‐10, Handpi). The electrical output of the piezoelectric transducer was measured using oscilloscopes (RIGOL DHO1104 or Analog Discovery 2). All vibration tests were performed using a programmable electrodynamic shaker (VE‐5120 M), which provides controlled excitation under user‐specified frequencies and amplitudes. A BLE sniffer (NRF52840 Dongle) was employed to capture the BLE advertising packets transmitted by the BLE module. The collected data were analyzed using Wireshark in combination with custom Python scripts for BLE MAC address filtering and packet parsing. In addition, a PC‐based Python program was developed to visualize the reconstructed vibration waveforms in real time.

## Author Contributions

G.H. conceived the idea, secured the funding support, and supervised the project. Y.L. and Y.Z (**Ye Zhang**) designed the experiments, conducted the tests, and collected the data. Y.L. prepared the figures and drafted the original manuscript. H.T., Y.Z. (**Yaozi Zheng**), and Y.W. supported the experiments and figure preparation. Y.L. designed and developed the printed circuit boards. H.T. assisted in the circuit design. G.H., J.W., and C.L. revised and polished the manuscript. C.Z., L.D, and D.Y. participated in the research and investigation process.

## Conflicts of Interest

The authors declare no conflicts of interest.

## Supporting information




**Supporting File 1**: advs76967‐sup‐0001‐SuppMat.pdf.

**Supporting File 2**: advs76967‐sup‐0002‐VideoS1.mp4.

**Supporting File 3**: advs76967‐sup‐0003‐VideoS2.mp4.

**Supporting File 4**: advs76967‐sup‐0004‐VideoS3.mp4.

## Data Availability

The data that support the findings of this study are available from the corresponding author upon reasonable request.
