# Supplementary material for: Sustained Self‐Powered Real‐Time Vibration Monitoring Through Integrated Nonlinear Harvesting and Energy‐Aware Wireless Sensing
Source: Adv Sci (Weinh). 2026 Aug 5;13(60):e76967. doi: 10.1002/advs.76967 (PMC13440374; doi:10.1002/advs.76967)
Supplement: Supplementary file 1 [file ADVS-13-e76967-s002.pdf]

Supporting Information

List of Figures

Figure S1. Structural components of the integrated self-powered sensing system.

Figure S2. Experimental setup and geometric dimensions of the energy harvesting unit.

Figure S3. Experimental platform for mechanical characterization.

Figure S4. Force–displacement curves under different magnet configurations.

Figure S6. Raw time-domain voltage signals of the QZS harvester during frequency sweeping tests.

Figure S7. Raw time-domain voltage signals of the QZS harvester during upward frequency sweeps under varying excitation amplitudes. (A) 0.12g, (B) 0.14g, (C) 0.16g.

Figure S8. Raw time-domain voltage signals of the traditional linear harvester during frequency sweeps under varying excitation amplitudes. (A) 0.12g, (B) 0.14g, (C) 0.16g.

Figure S9. Raw voltage responses of the QZS harvester at a constant acceleration amplitude of 0.16g under different excitation frequencies: (A) 6 Hz, (B) 6.25 Hz, (C) 6.5 Hz, (D) 6.75 Hz, (E) 7 Hz, (F) 7.25 Hz, (G) 7.5 Hz, (H) 7.75 Hz, (I) 8 Hz.

Figure S10. Fast Fourier Transform (FFT) spectra of the raw voltage responses of the QZS harvester at a constant acceleration amplitude of 0.16g under different excitation frequencies: (A) 6 Hz, (B) 6.25 Hz, (C) 6.5 Hz, (D) 6.75 Hz, (E) 7 Hz, (F) 7.25 Hz, (G) 7.5 Hz, (H) 7.75 Hz, (I) 8 Hz.

Figure S11. Time-domain voltage responses and corresponding frequency spectra of the linear cantilever beam at its resonant frequency of 9.3 Hz.

Figure S12. Circuit schematic and printed circuit board (PCB) layout of the SECE interface.

Figure S13. Photographs of the custom-designed functional circuit modules.

Figure S14. Dynamic and spectral characterization of the QZS harvester without the SECE interface at an excitation frequency of 6.75 Hz under different acceleration amplitudes.

Figure S15. Dynamic and spectral characterization of the QZS harvester integrated with the SECE interface at an excitation frequency of 6.75 Hz under different acceleration amplitudes.

Figure S16. Experimental setup used for displacement measurement of the QZS harvester.

Figure S17. Comparison of the displacement responses between the linear and QZS harvesters.

Figure S18. Experimental setup used for power consumption analysis.

Figure S19. Current consumption profiles of the wireless sensing module under different supply voltages at a fixed sampling rate of 50 Sa/s: (A) 1.8 V, (B) 2.5 V, (C) 3.3 V, (D) 3.6 V.

Figure S20. Current consumption profiles of the wireless sensing module at a constant supply voltage of 1.8 V under different sampling rates: (A) 25 Sa/s, (B) 50 Sa/s, (C) 75 Sa/s, (D) 100 Sa/s.

Figure S21. Schematic illustration of the experimental platform for evaluating the self-powered wireless sensing system.

Figure S22. Photograph of the experimental testbed for the self-powered wireless vibration sensing system.
Figure S23. Flowchart illustrating the packet capture and data processing workflow at the receiving end.

List of Tables

Table S1. Detailed geometric and material properties of the components used in developing the self-powered sensing system.
Table S2. Comparison of continuous operation capability and effective sampling rates among representative self-powered IoT sensing nodes.

List of Notes

Supplementary Note S1. Long-Term Cyclic Stability and Temperature-Dependent Robustness of the QZS Harvester.

List of Videos

Video S1. Comparison of vibration amplitudes: QZS and linear harvesters.
Video S2. Comparison of the charging performance: full-bridge rectifier and SECE circuits.
Video S3. Experiment validation of self-powered real-time sensing system.

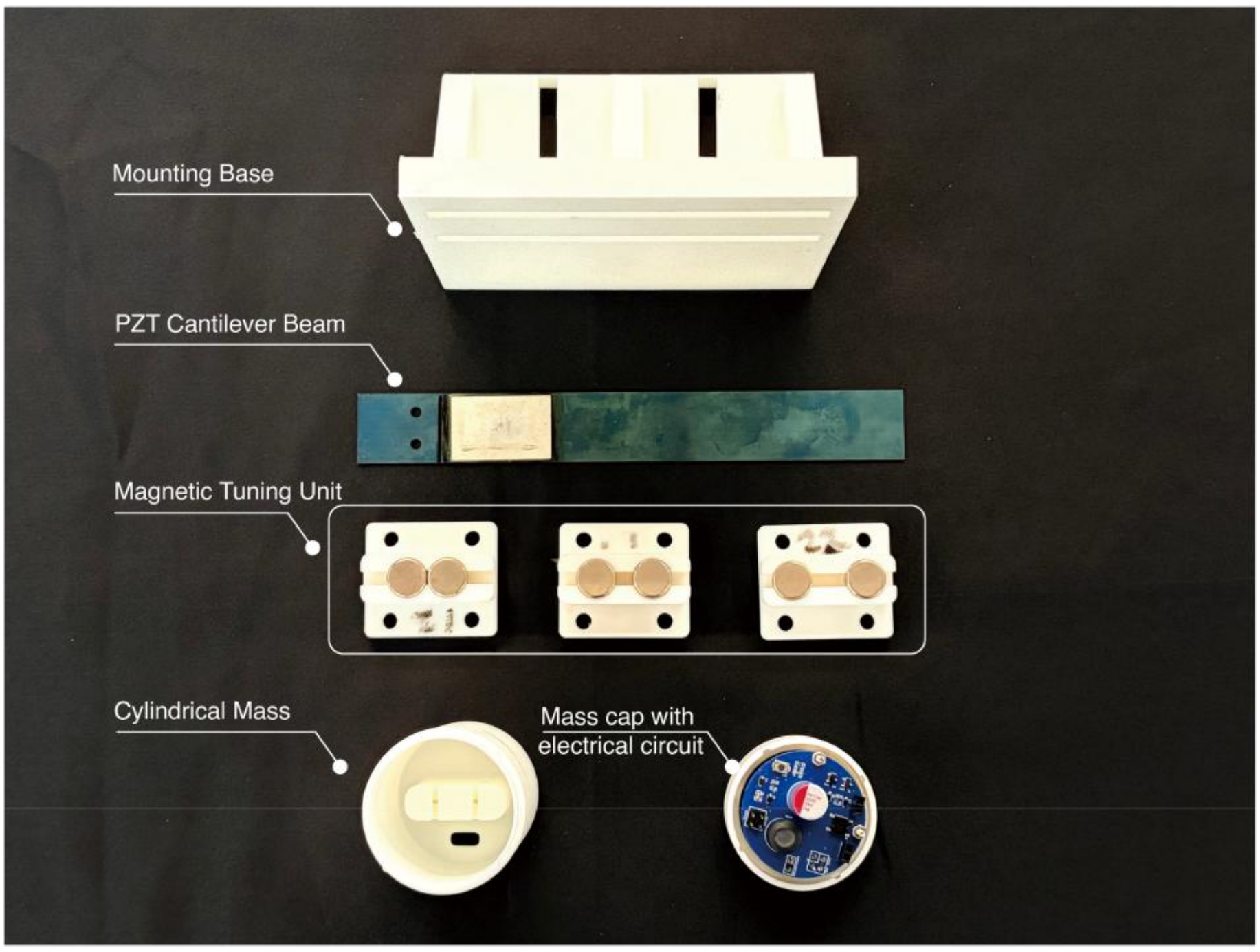

Figure S1. Structural components of the integrated self-powered sensing system.

A

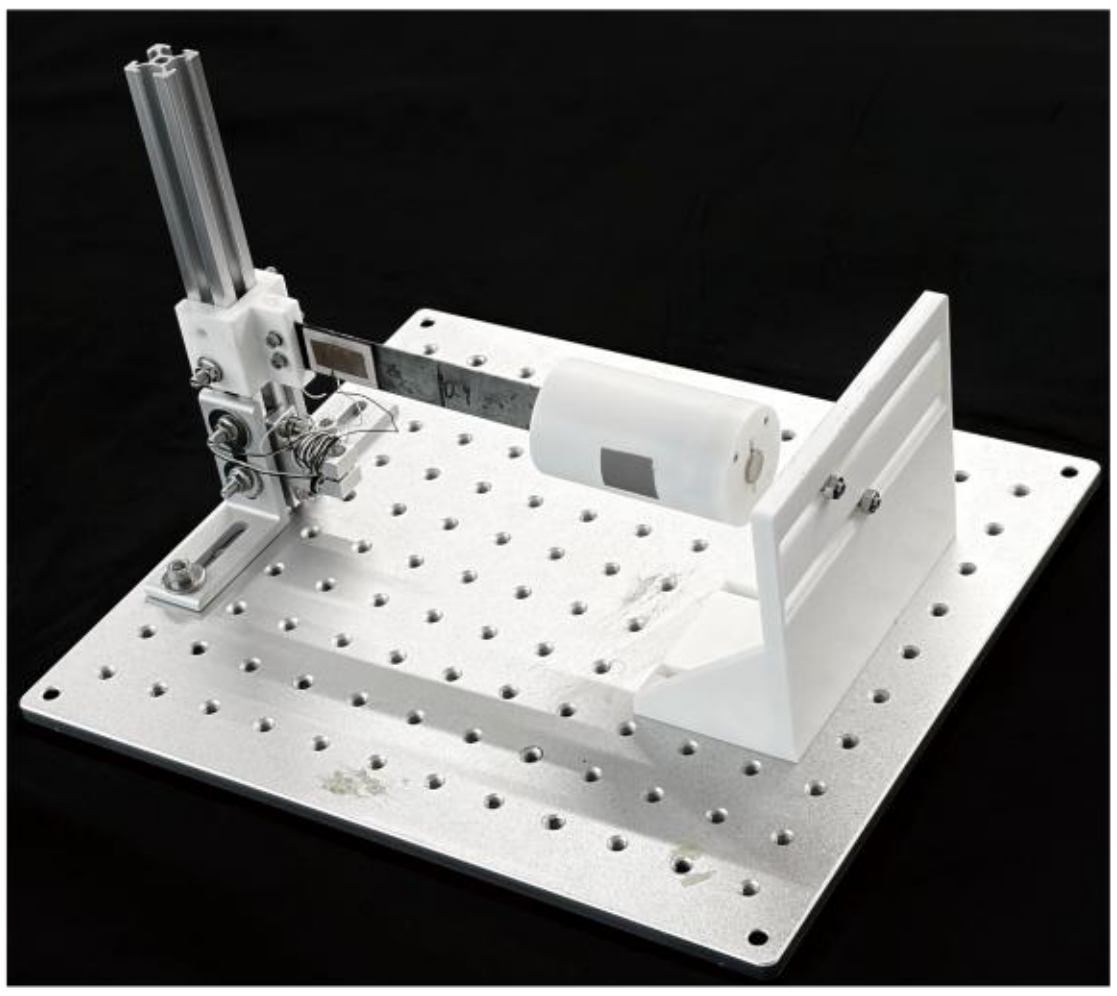

B

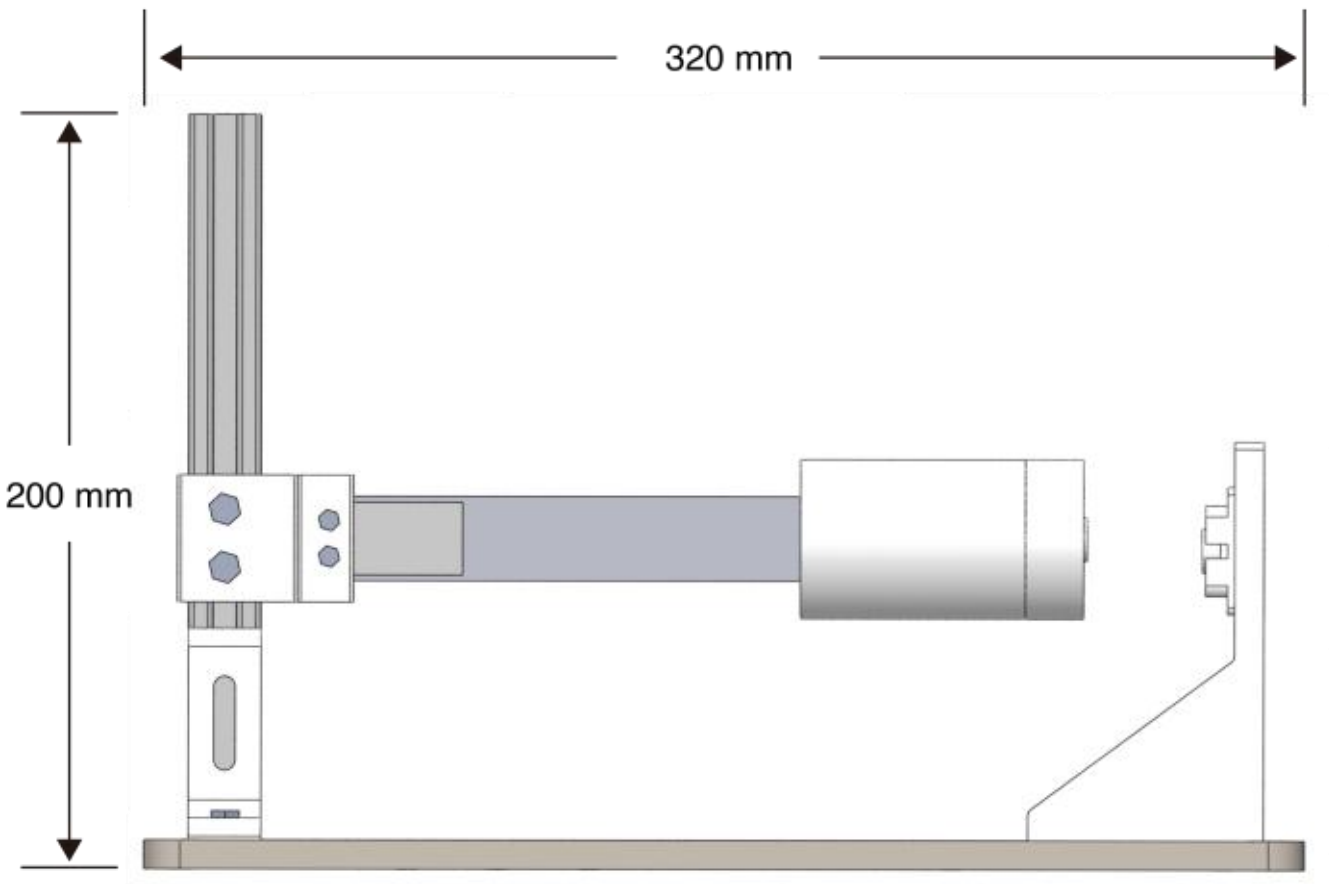

Figure S2. Experimental setup and geometric dimensions of the energy harvesting unit.
(A) Photograph of the fully assembled prototype mounted on an aluminum-profile support frame.
(B) Schematic illustration showing the overall dimensions and magnet spatial configuration of the QZS piezoelectric energy harvester.

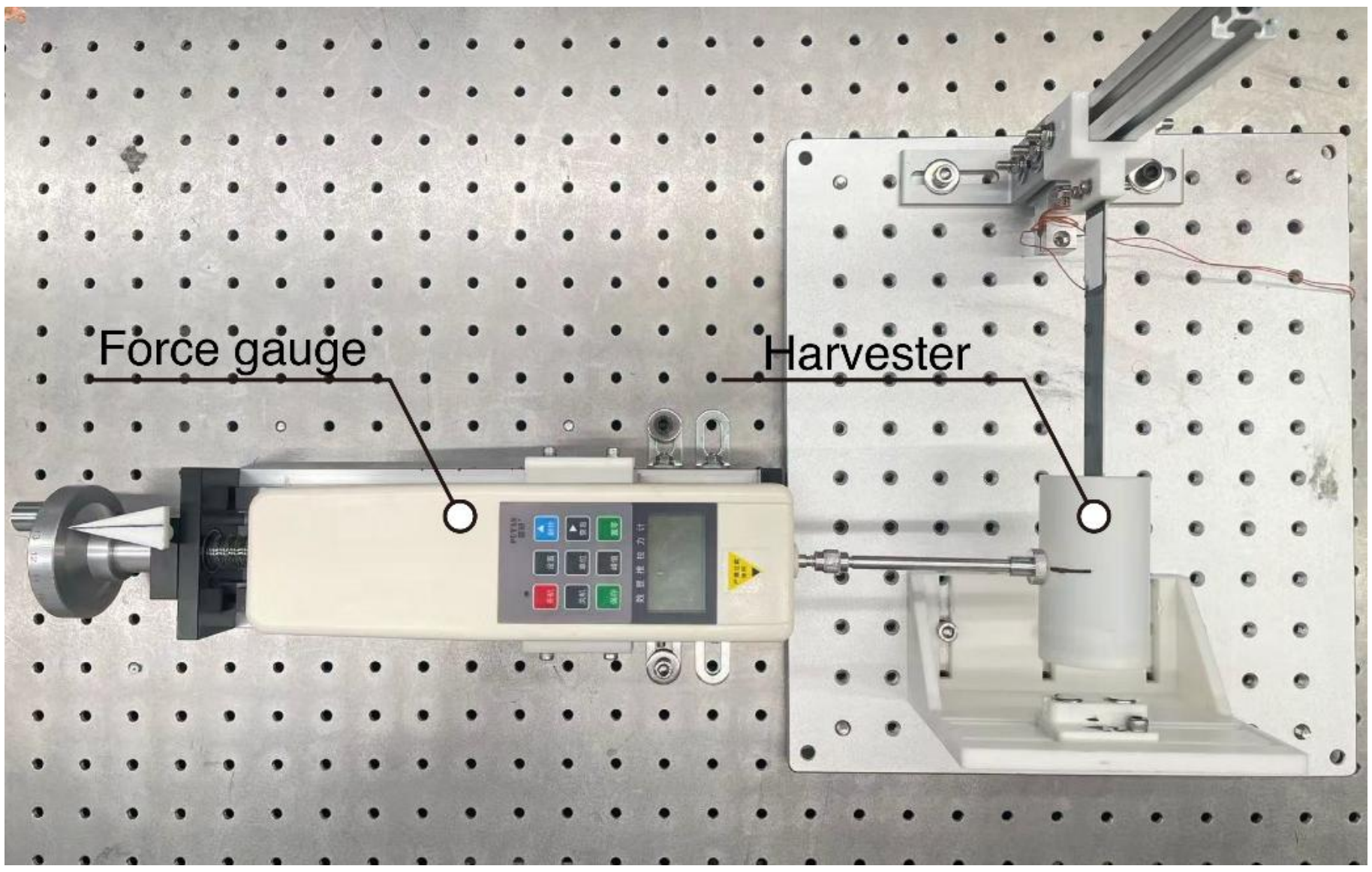

Figure S3. Experimental platform for mechanical characterization.

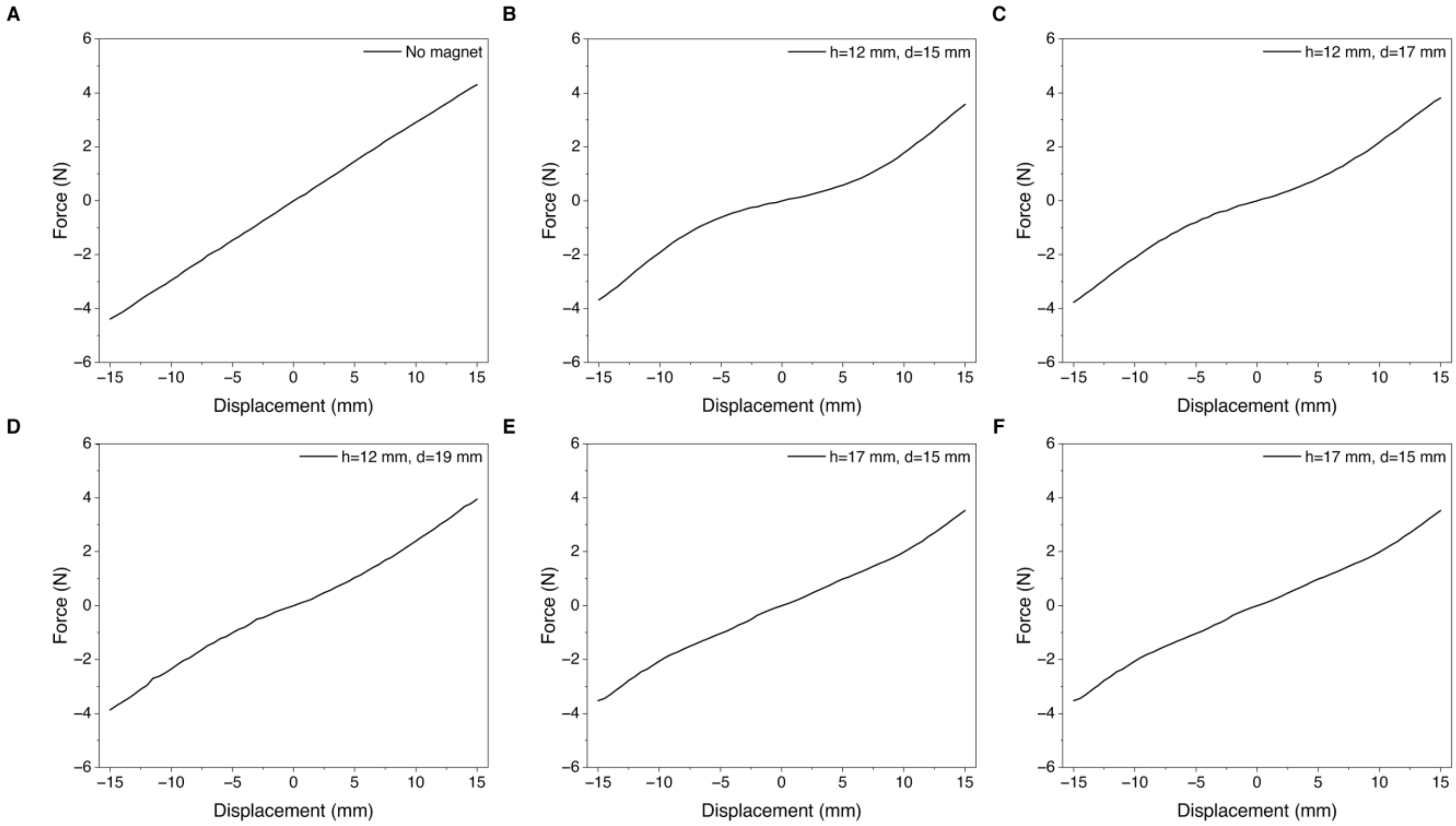

Figure S4. Force–displacement curves under different magnet configurations.

(A) Linear force–displacement relationship of the cantilever beam without magnets.

(B-F) Measured force–displacement curves under different horizontal magnet distances d and vertical spacings h: (B) h = 12 mm, d = 15 mm; (C) h = 12 mm, d = 17 mm; (D) h = 12 mm, d = 19 mm; (E) h = 17 mm, d = 15 mm; (F) h = 17 mm, d = 15 mm.

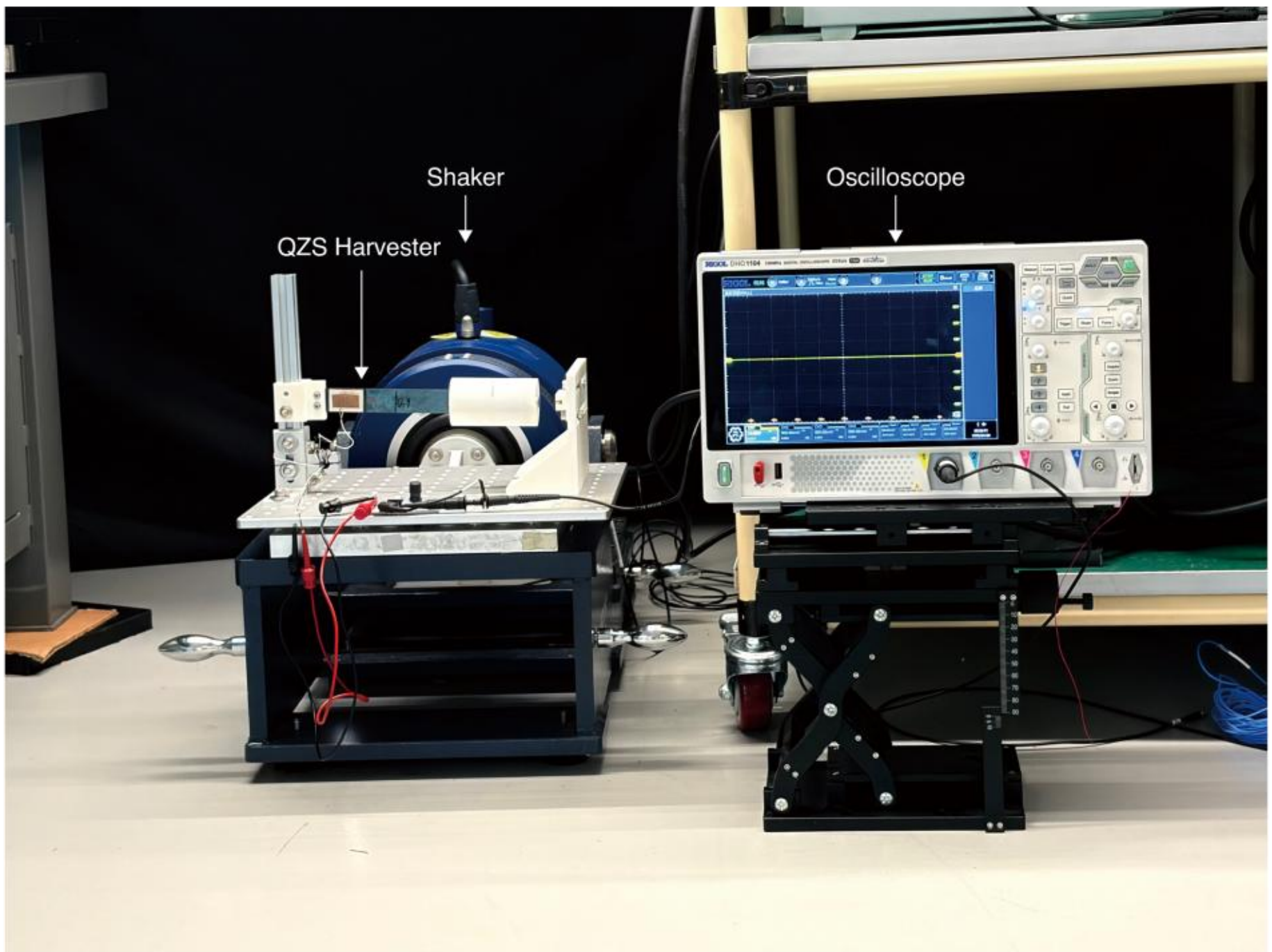

Figure S5. Experimental setup used for dynamic and electrical characterization.

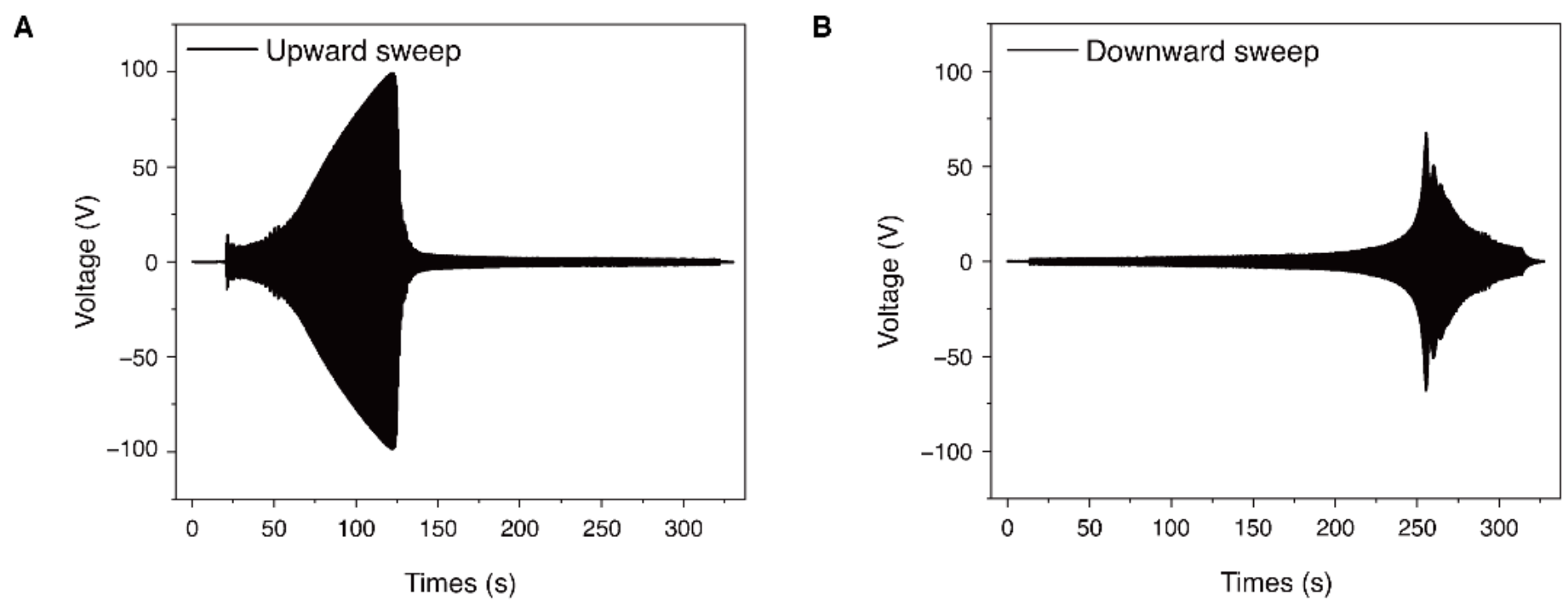

Figure S6. Raw time-domain voltage signals of the QZS harvester during frequency sweeping tests.
(A) Upward sweep: Raw voltage response as the frequency increases, showing the jump-down behavior at the bifurcation point.
(B) Downward sweep: Raw voltage response as the frequency decreases, illustrating the jump-up transition and narrower high-amplitude region.

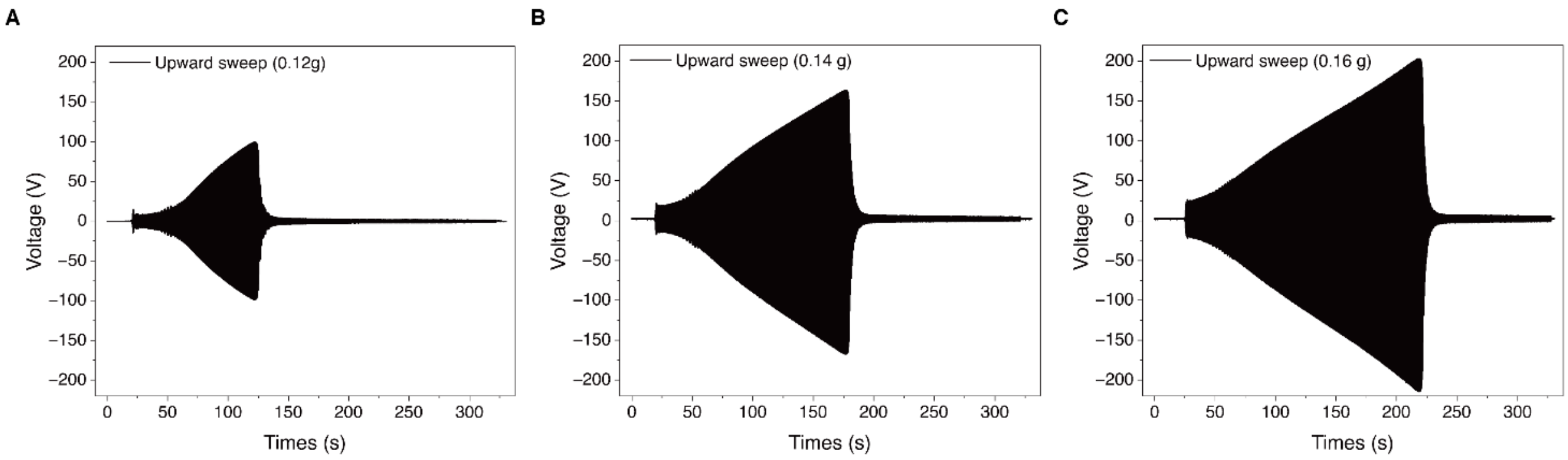

Figure S7. Raw time-domain voltage signals of the QZS harvester during upward frequency sweeps under varying excitation amplitudes. (A) 0.02g, (B) 0.04g, (C) 0.06g.

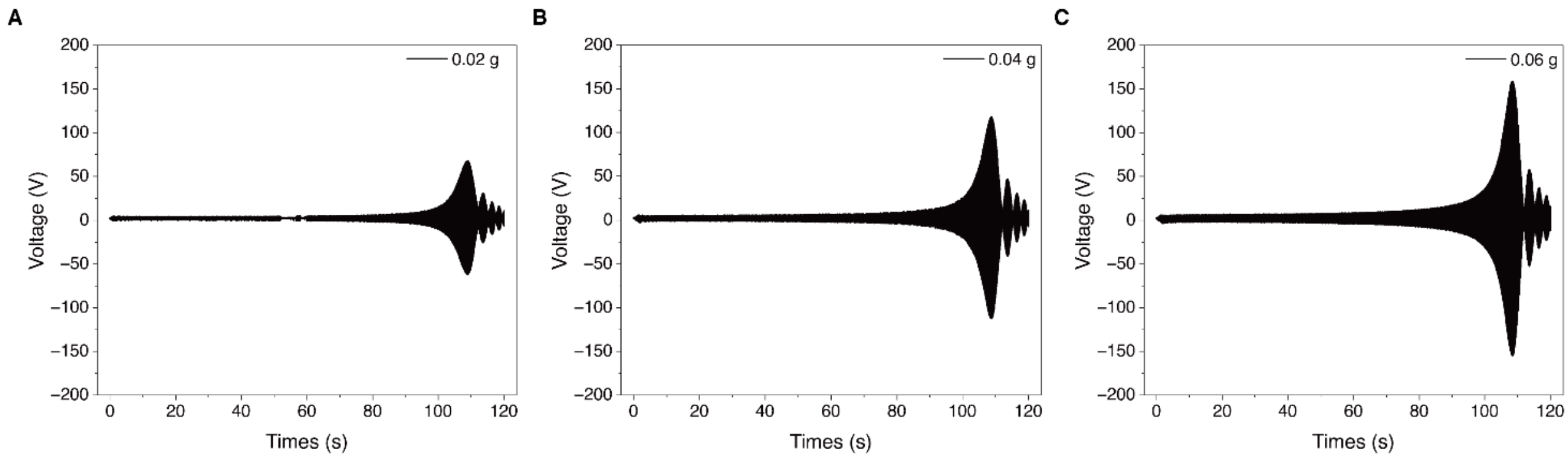

Figure S8. Raw time-domain voltage signals of the traditional linear harvester during frequency sweeps under varying excitation amplitudes. (A) 0.02g, (B) 0.04g, (C) 0.06g.

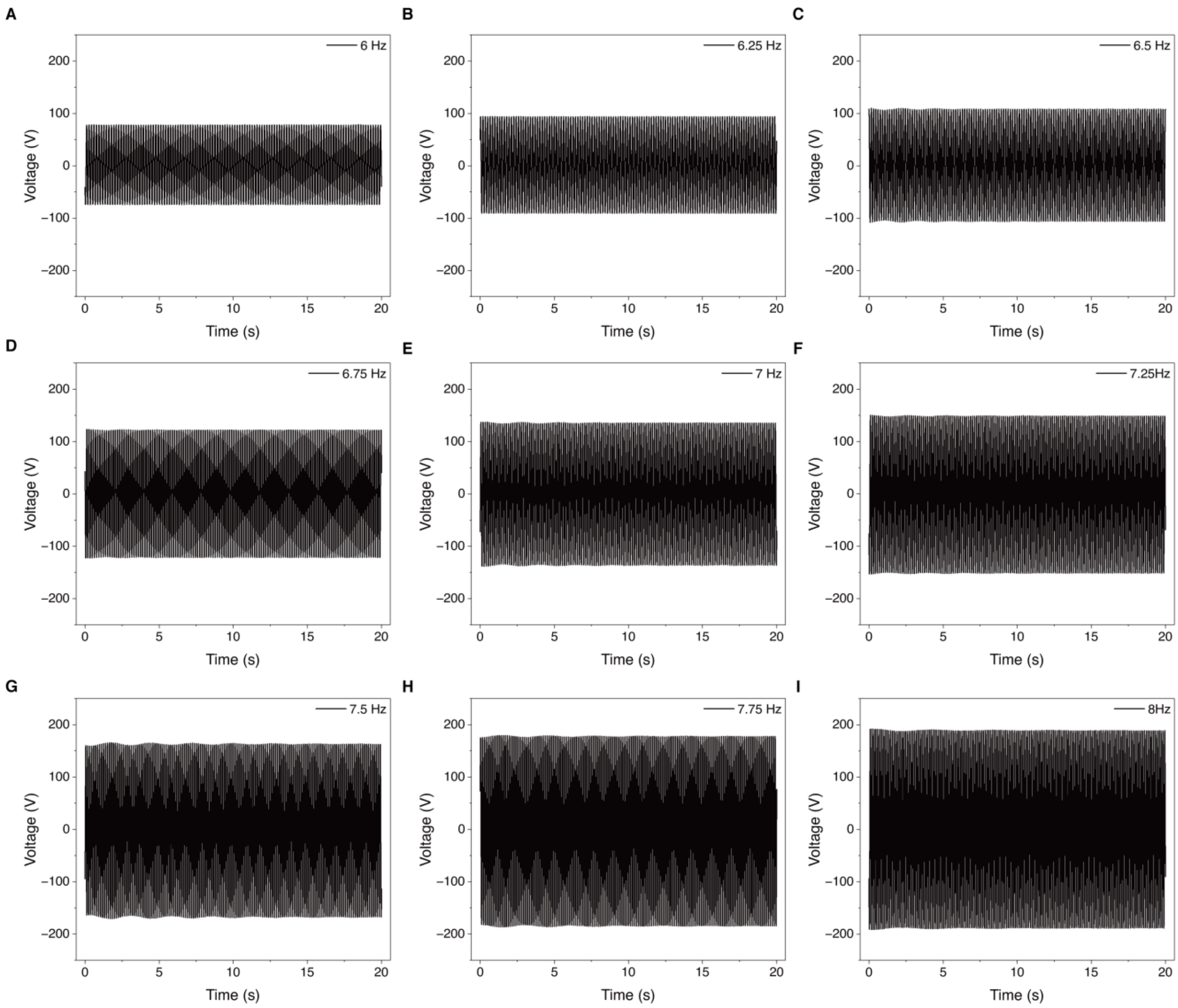

Figure S9. Raw voltage responses of the QZS harvester at a constant acceleration amplitude of 0.16g under different excitation frequencies: (A) 6 Hz, (B) 6.25 Hz, (C) 6.5 Hz, (D) 6.75 Hz, (E) 7 Hz, (F) 7.25 Hz, (G) 7.5 Hz, (H) 7.75 Hz, (I) 8 Hz.

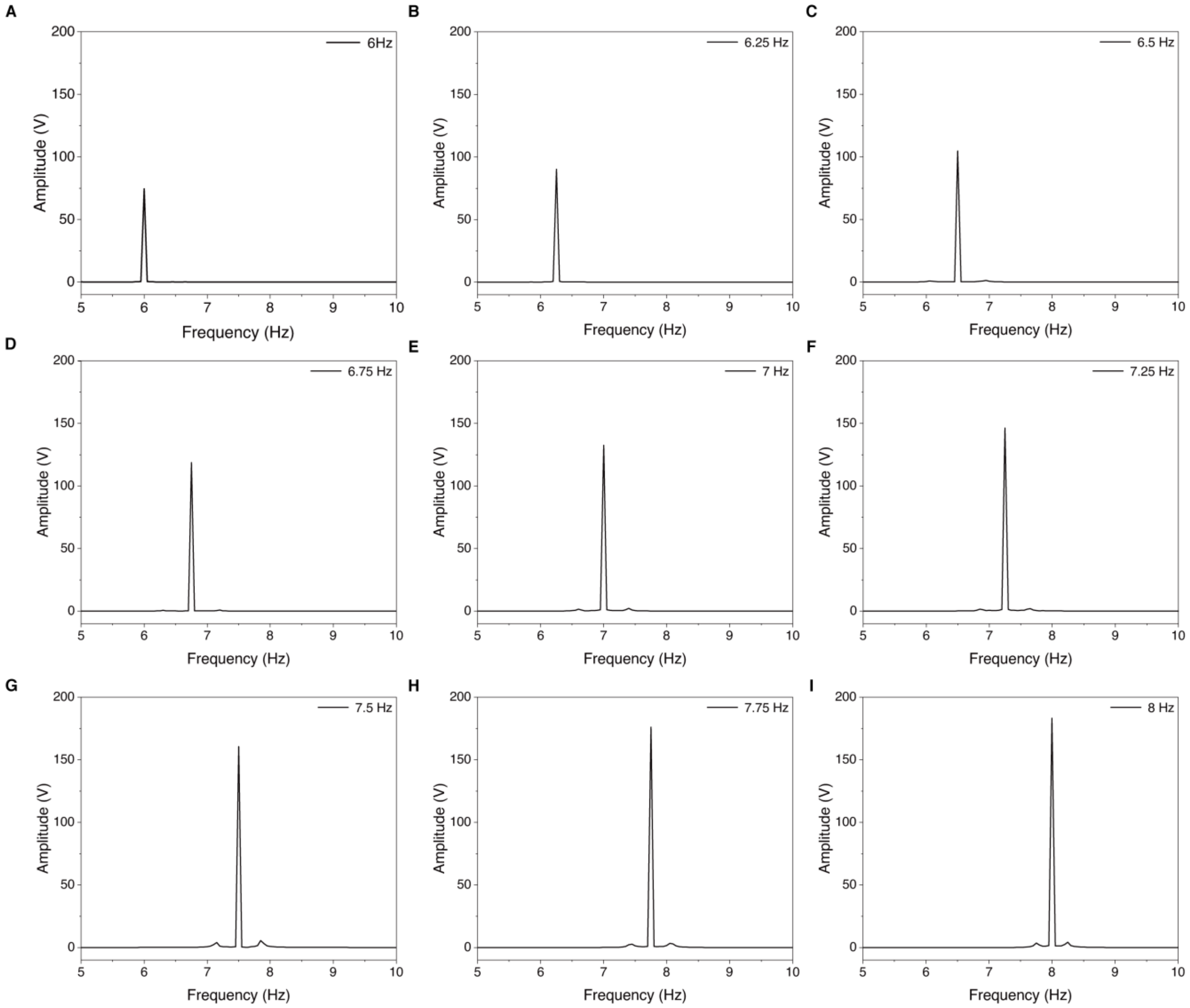

Figure S10. Fast Fourier Transform (FFT) spectra of the raw voltage responses of the QZS harvester at a constant acceleration amplitude of 0.16g under different excitation frequencies: (A) 6 Hz, (B) 6.25 Hz, (C) 6.5 Hz, (D) 6.75 Hz, (E) 7 Hz, (F) 7.25 Hz, (G) 7.5 Hz, (H) 7.75 Hz, (I) 8 Hz.

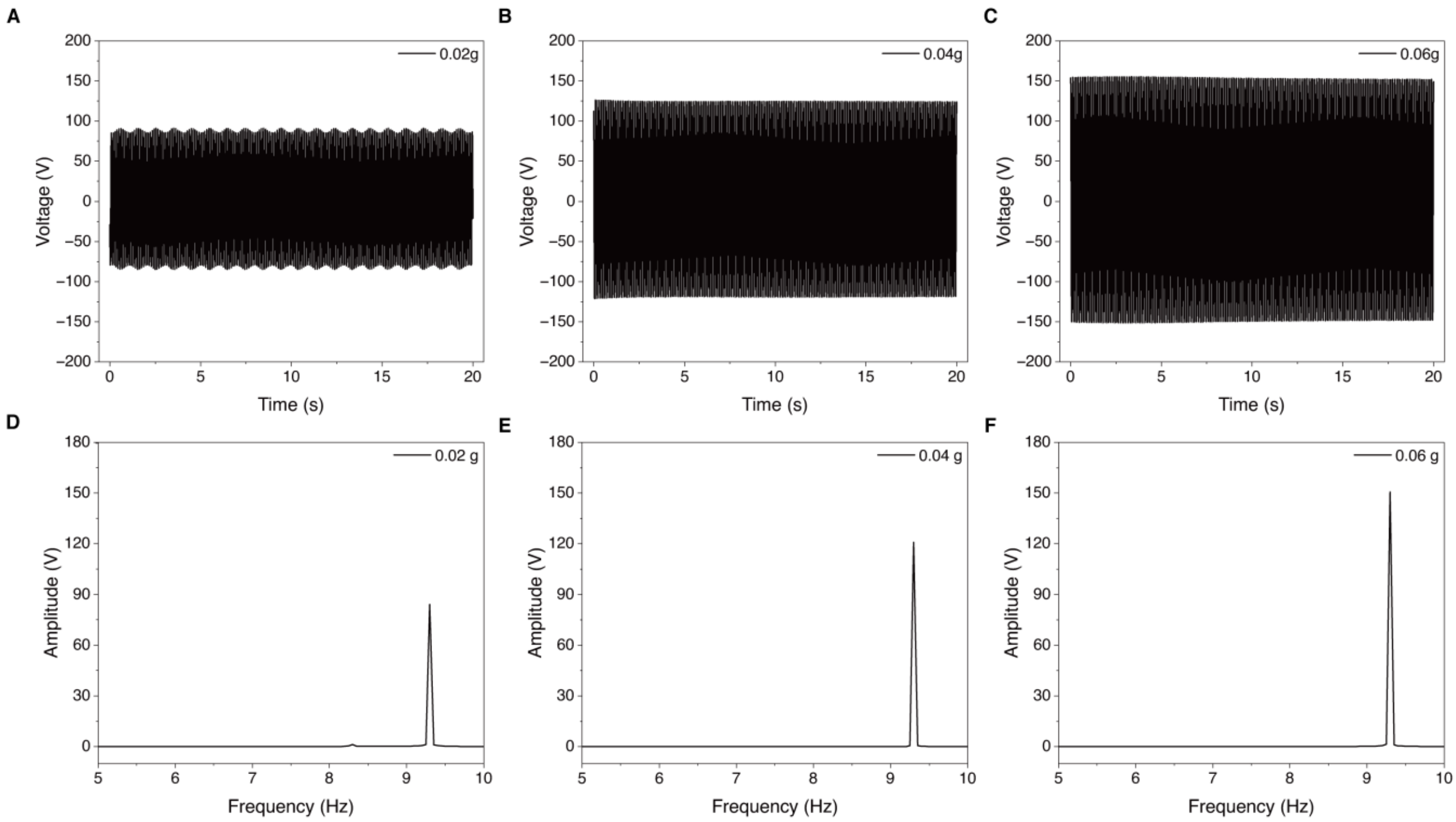

Figure S11. Time-domain voltage responses and corresponding frequency spectra of the linear cantilever beam at its resonant frequency of 9.3 Hz.

(A-C) Raw time-domain voltage outputs under constant acceleration amplitudes of (A) 0.02g, (B) 0.04g, and (C) 0.06g.

(D-F) Corresponding fast Fourier Transform (FFT) spectra for excitation accelerations of (A) 0.02g, (B) 0.04g, and (C) 0.06g, respectively.

A

B

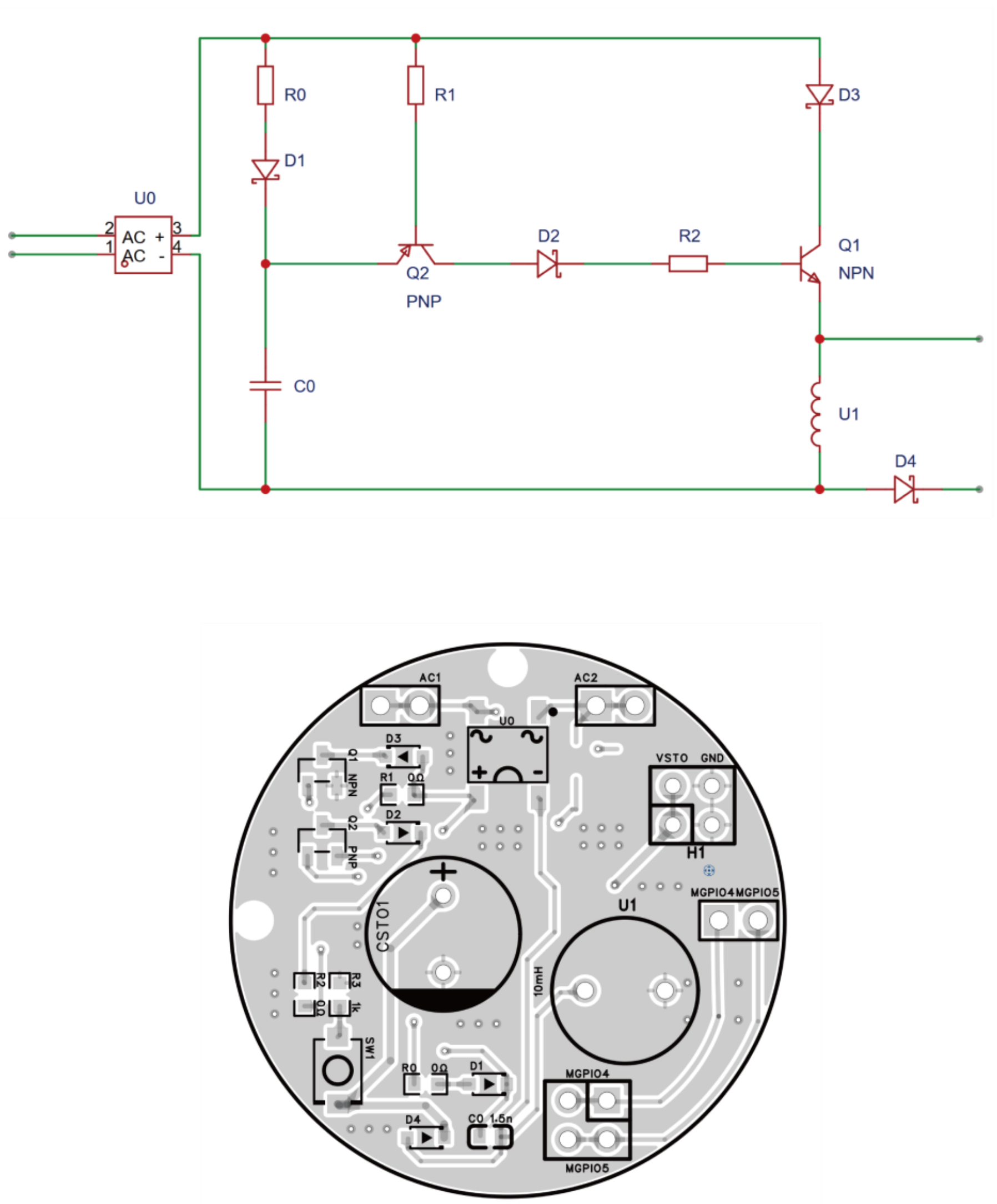

Figure S12. Circuit schematic and printed circuit board (PCB) layout of the SECE interface.
(A) Schematic diagram of the synchronous electric charge extraction (SECE) circuit.
(B) Top view of the custom-designed circular PCB for the SECE interface.

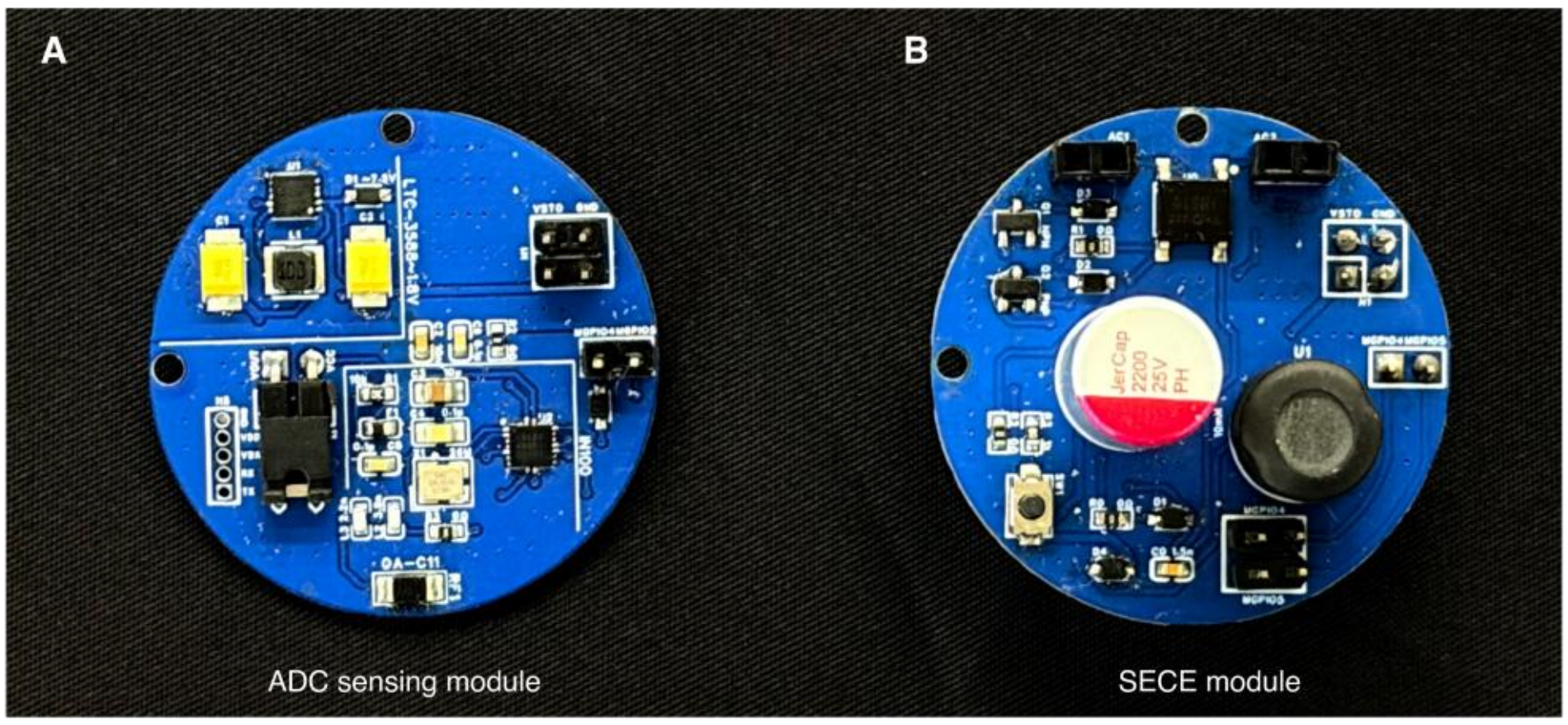

Figure S13. Photographs of the custom-designed functional circuit modules.
(A) ADC sensing module.
(B) SECE module.

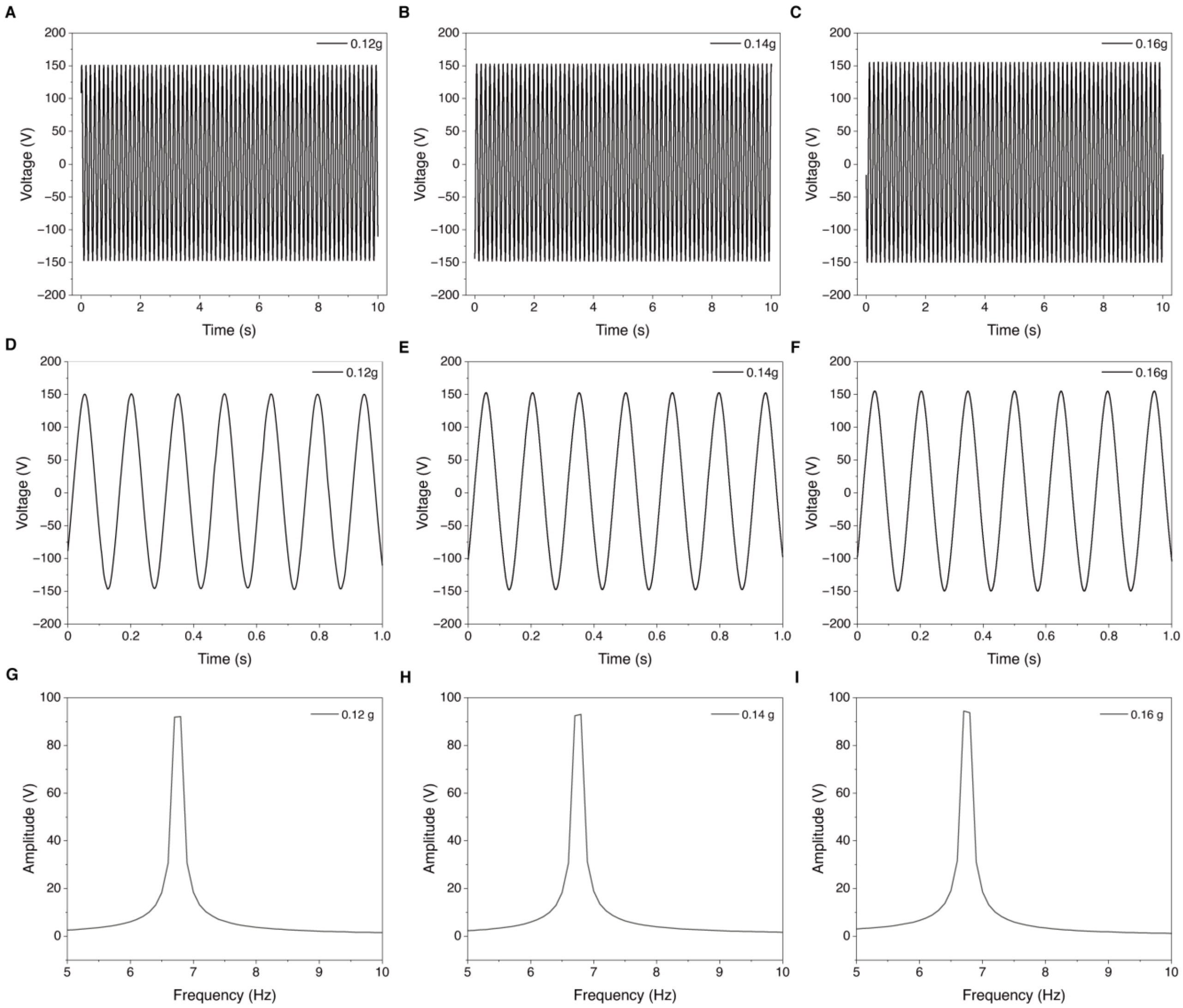

Figure S14. Dynamic and spectral characterization of the QZS harvester without the SECE interface at an excitation frequency of 6.75 Hz under different acceleration amplitudes.

(A-C) Long-duration time-domain voltage signals recorded over 10 seconds under acceleration amplitudes of (A) 0.12g, (B) 0.14g, and (C) 0.16g using a standard AC interface.

(D-F) Enlarged short-duration voltage waveforms corresponding to (A–C), showing the detailed sinusoidal responses.

(G-I) Corresponding fast FFT spectra, showing the dominant frequency component at 6.75 Hz.

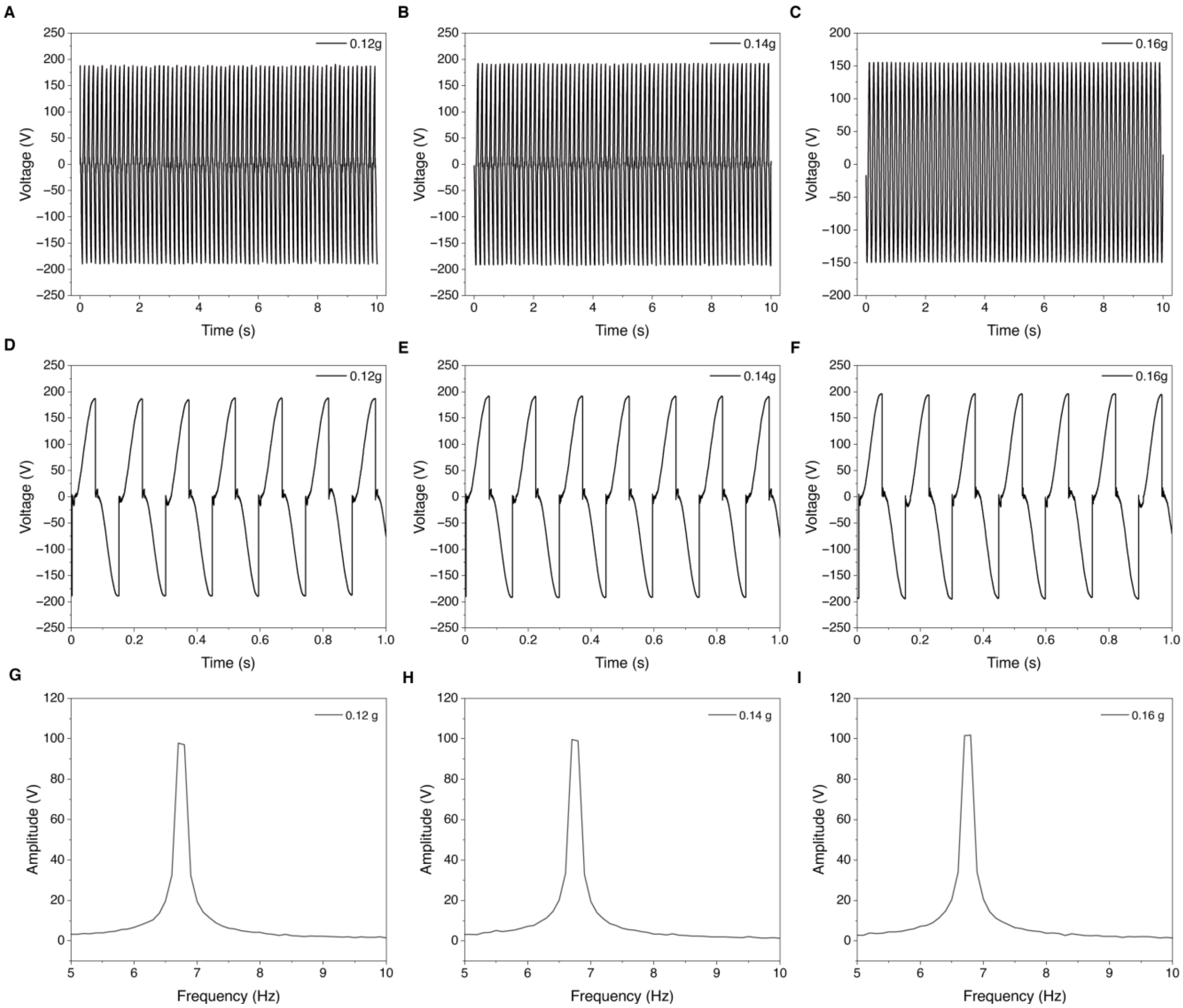

Figure S15. Dynamic and spectral characterization of the QZS harvester integrated with the SECE interface at an excitation frequency of 6.75 Hz under different acceleration amplitudes.

(A-C) Long-duration time-domain voltage signals recorded over 10 seconds under acceleration amplitudes of (A) 0.12g, (B) 0.14g, and (C) 0.16g.

(D-F) Enlarged short-duration voltage waveforms corresponding to (A–C), showing the detailed nonlinear voltage responses.

(G-I) Corresponding FFT spectra, showing the dominant frequency component at 6.75 Hz.

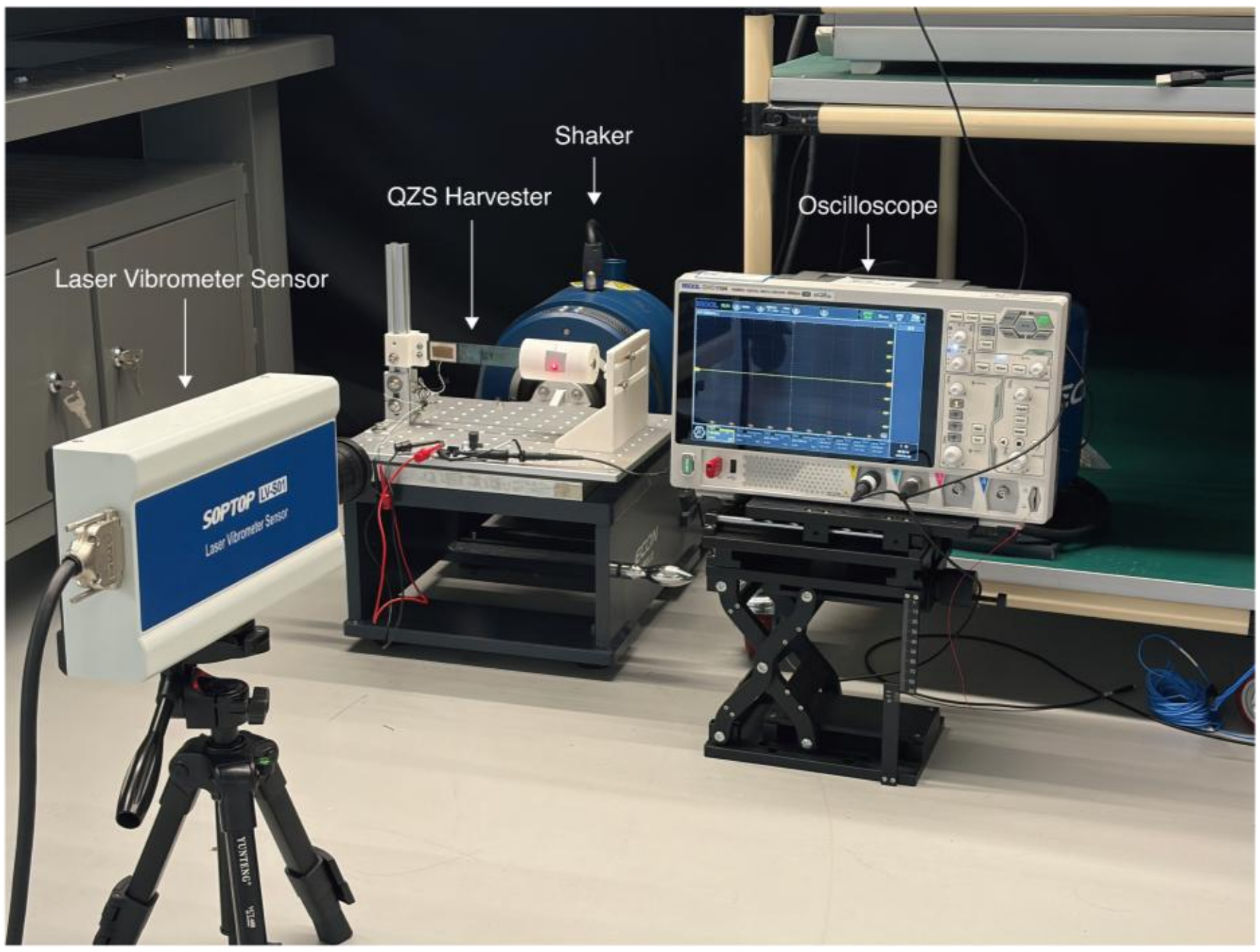

Figure S16. Experimental setup used for displacement measurement of the QZS harvester.

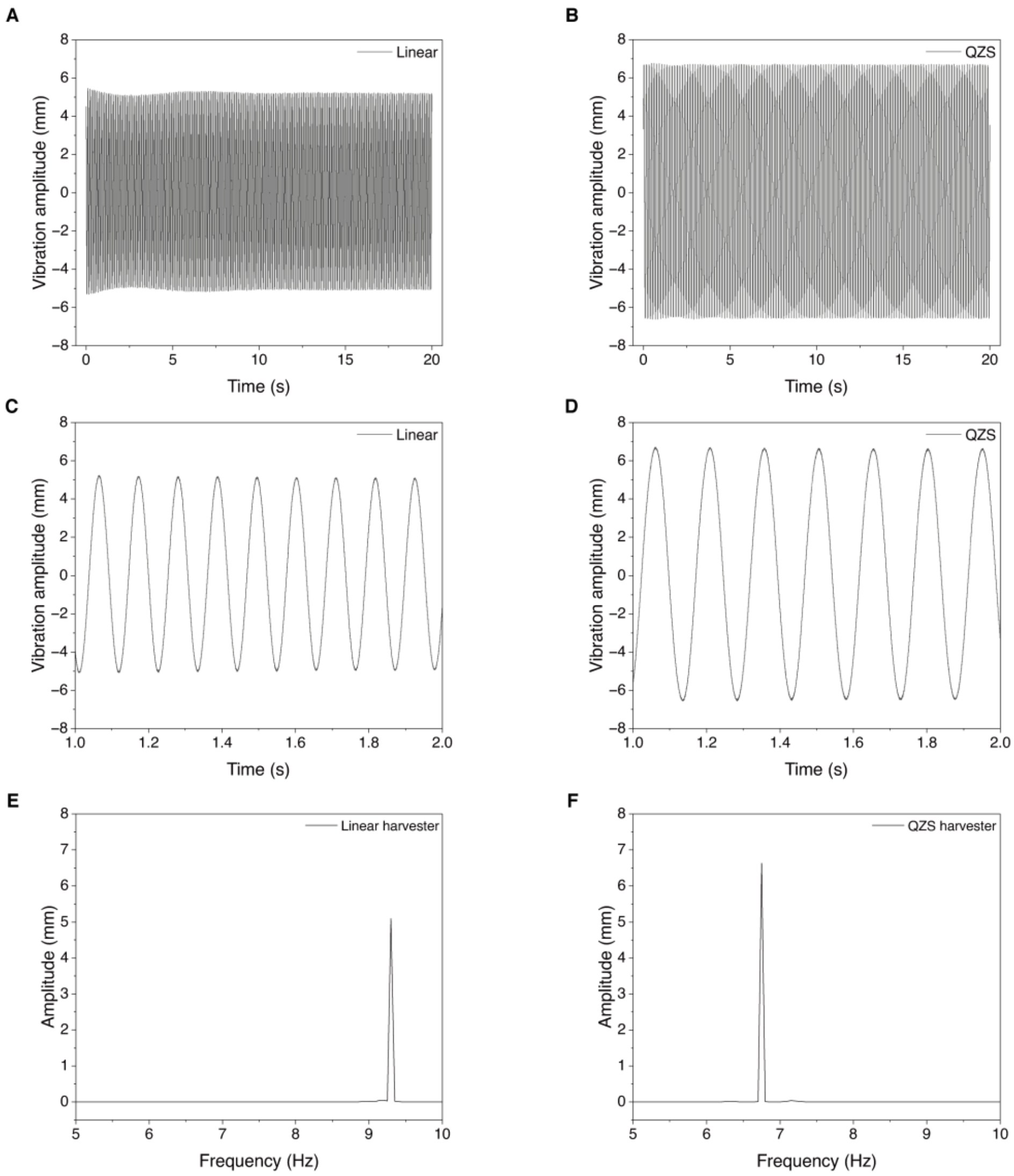

Figure S17. Comparison of the displacement responses between the linear and QZS harvesters.

(A-B) Long-duration time-domain displacement responses of the (A) linear harvester and (B) QZS harvester.

(C-D) Enlarged displacement waveforms of the (C) linear harvester and (D) QZS harvester.

(E-F) Corresponding FFT spectra of the displacement responses, showing the resonant peaks at (E) 9.3 Hz for the linear harvester and (F) 6.75 Hz for the QZS harvester.

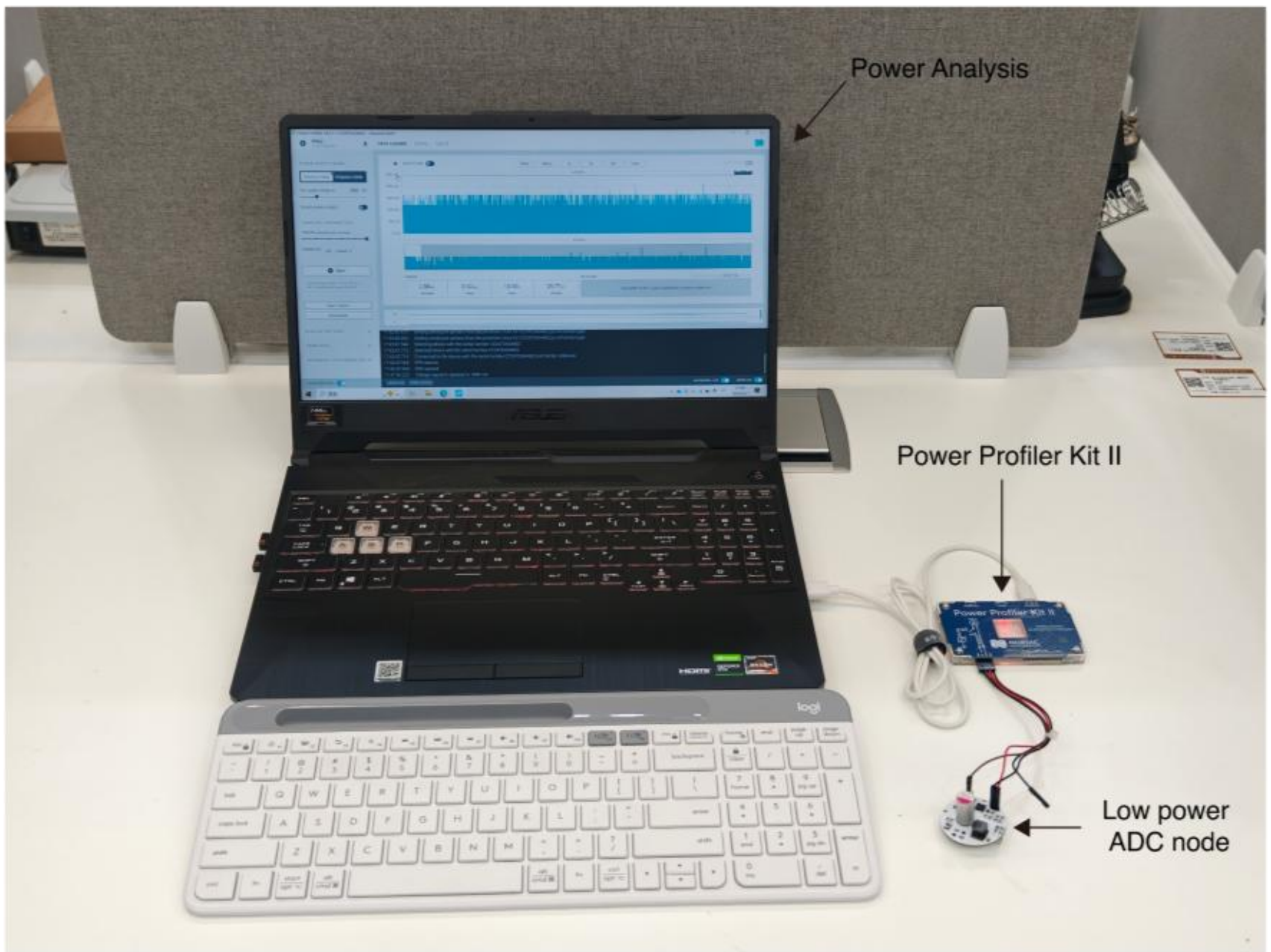

Figure S18. Experimental setup used for power consumption analysis.

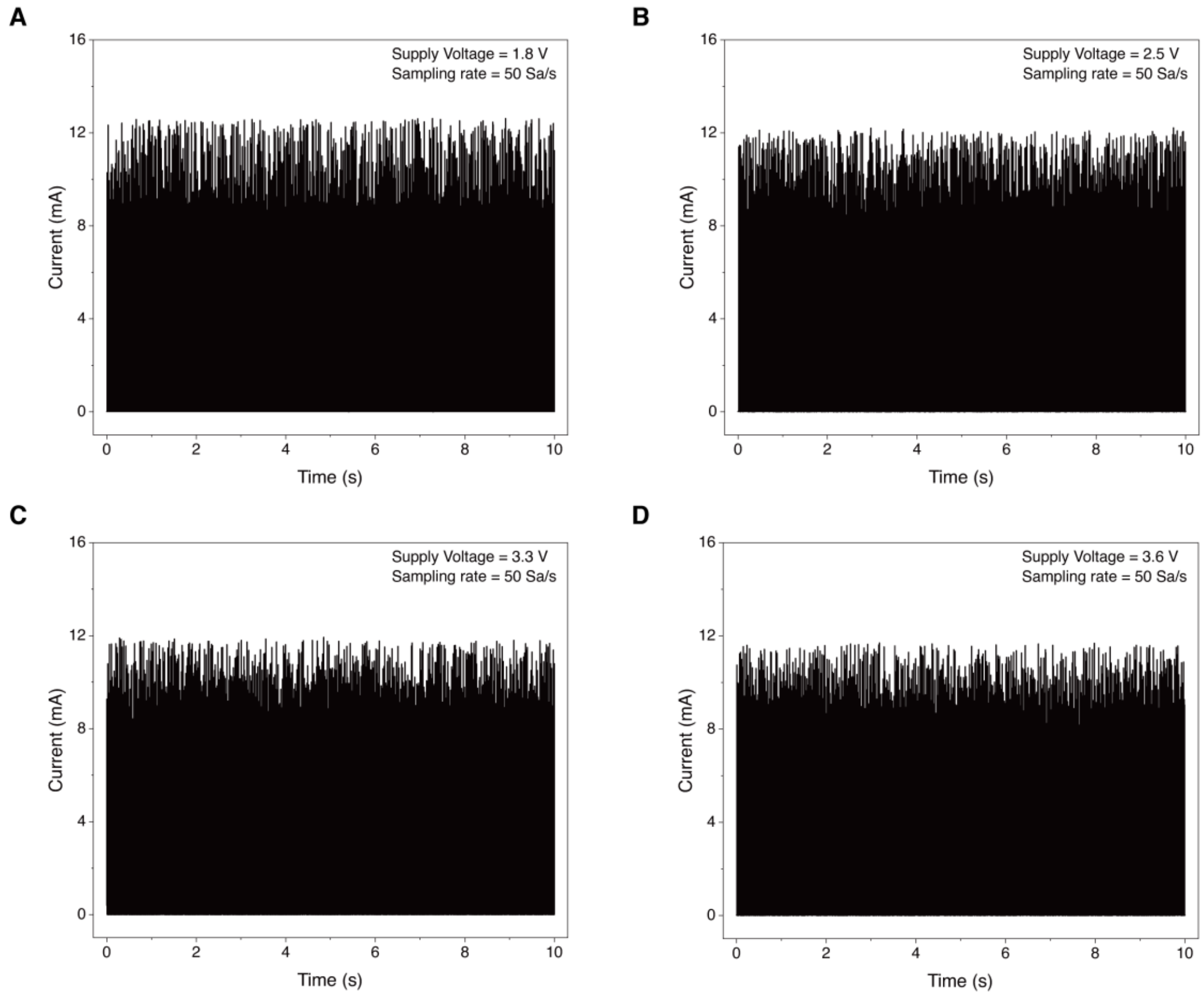

Figure S19. Current consumption profiles of the wireless sensing module under different supply voltages at a fixed sampling rate of 50 Sa/s: (A) 1.8 V, (B) 2.5 V, (C) 3.3 V, (D) 3.6 V.

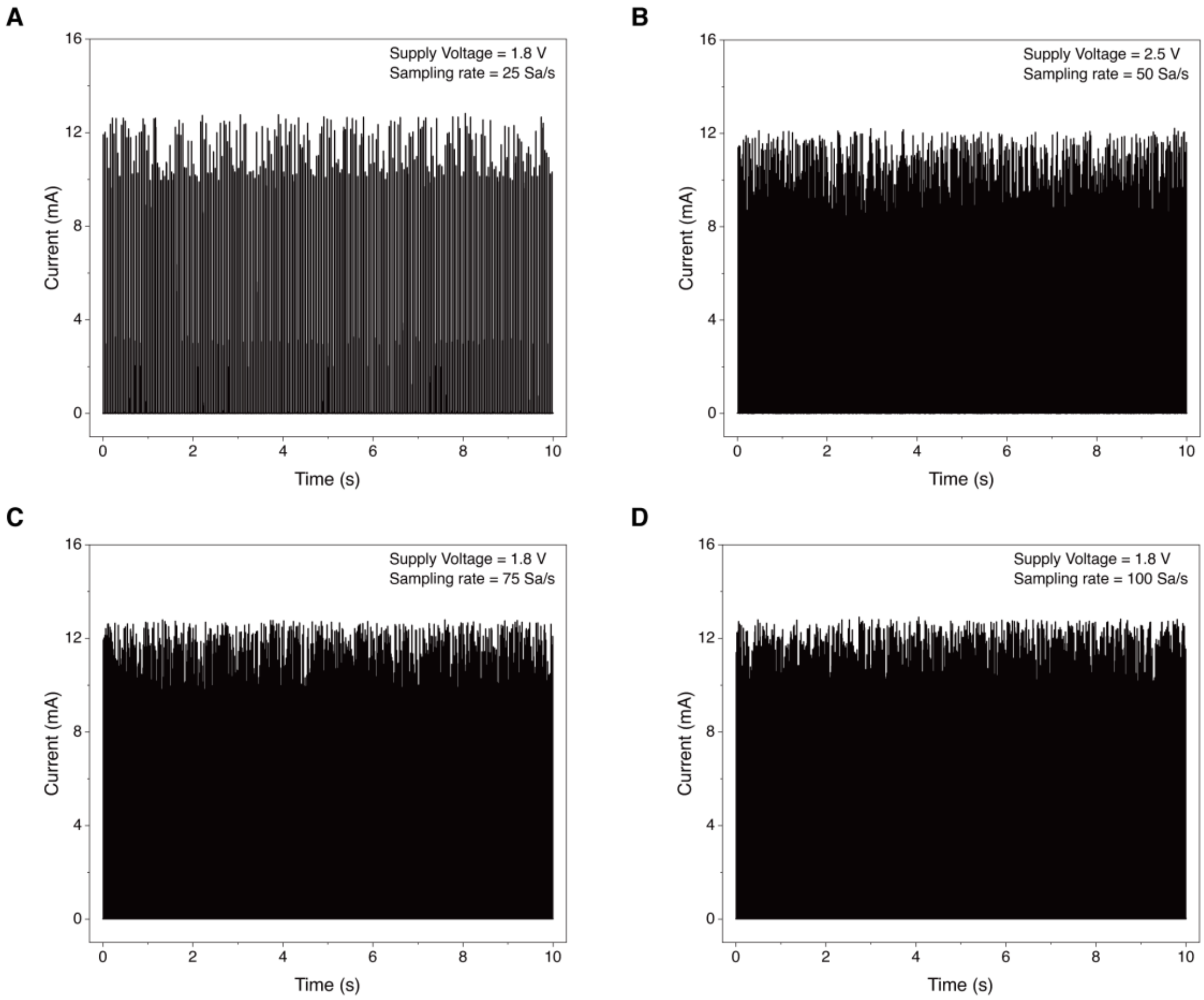

Figure S20. Current consumption profiles of the wireless sensing module at a constant supply voltage of 1.8 V under different sampling rates: (A) 25 Sa/s, (B) 50 Sa/s, (C) 75 Sa/s, (D) 100 Sa/s.

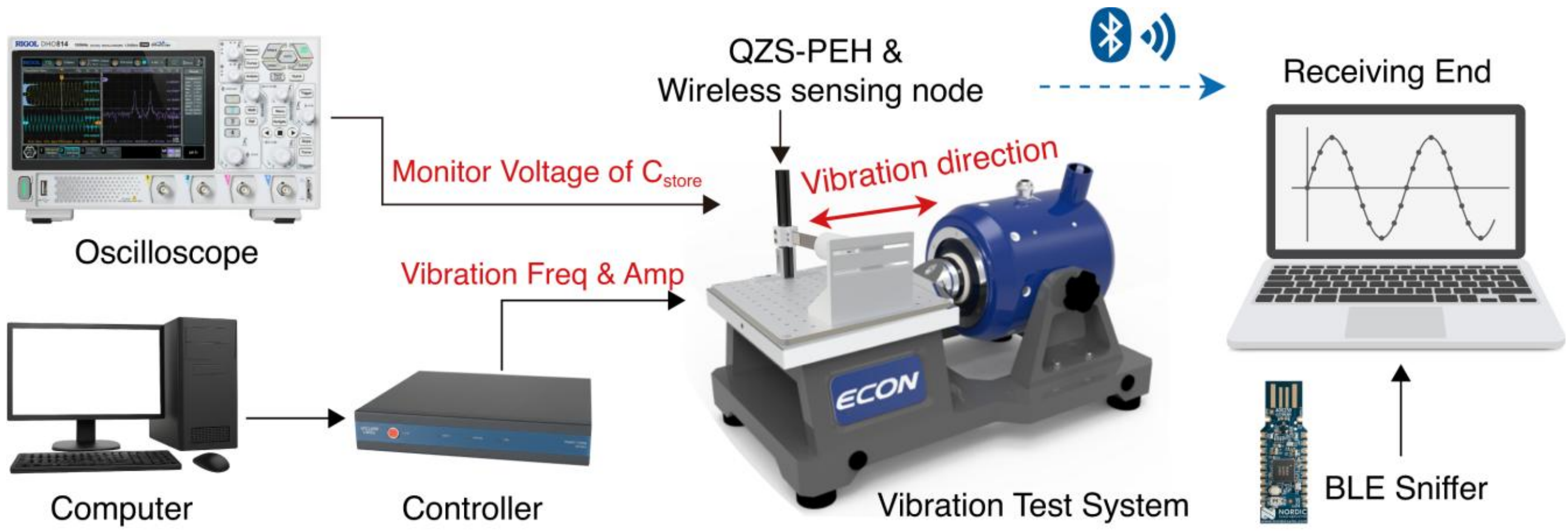

Figure S21. Schematic illustration of the experimental platform for evaluating the self-powered wireless sensing system.

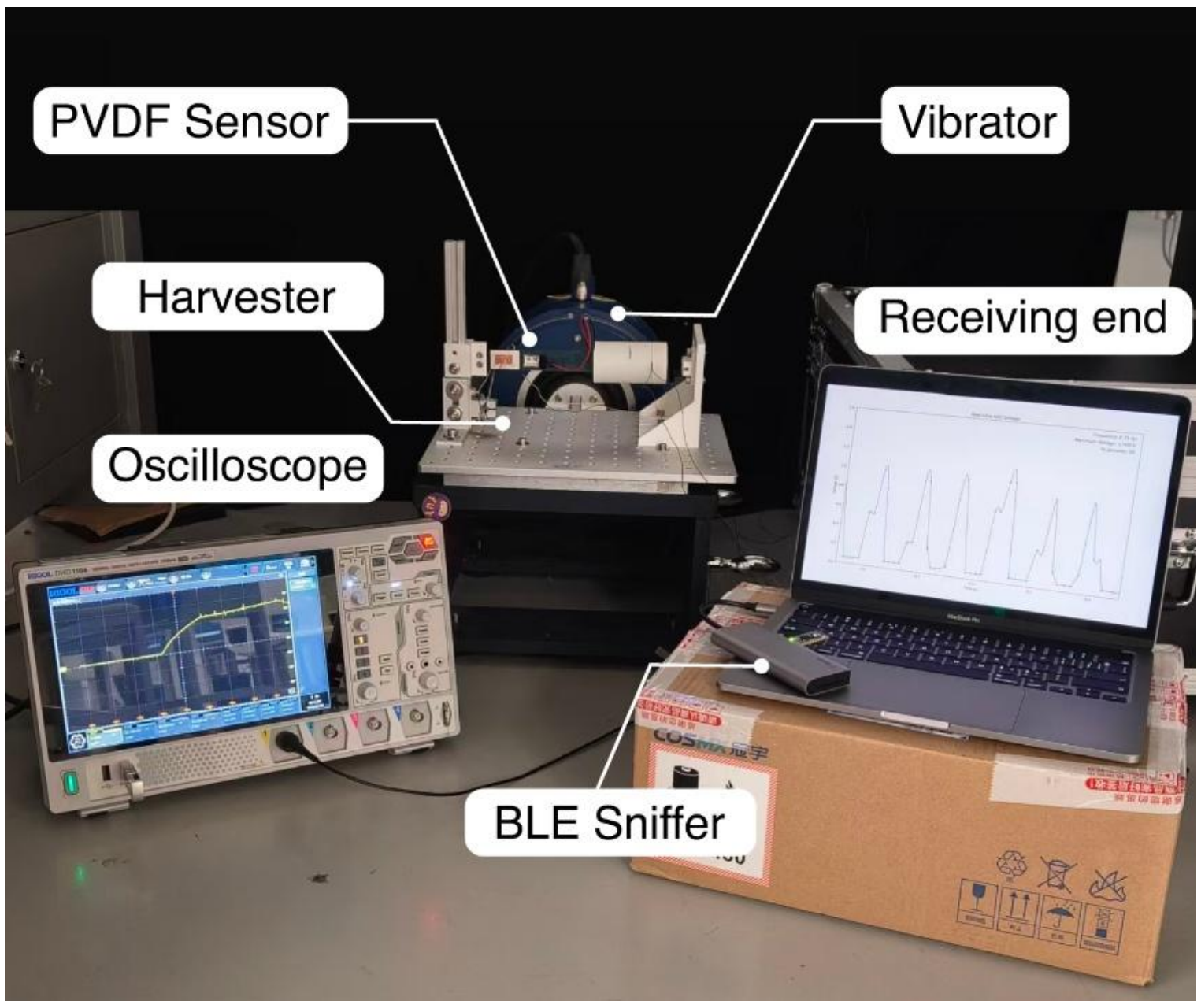

Figure S22. Photograph of the experimental testbed for the self-powered wireless vibration sensing system.

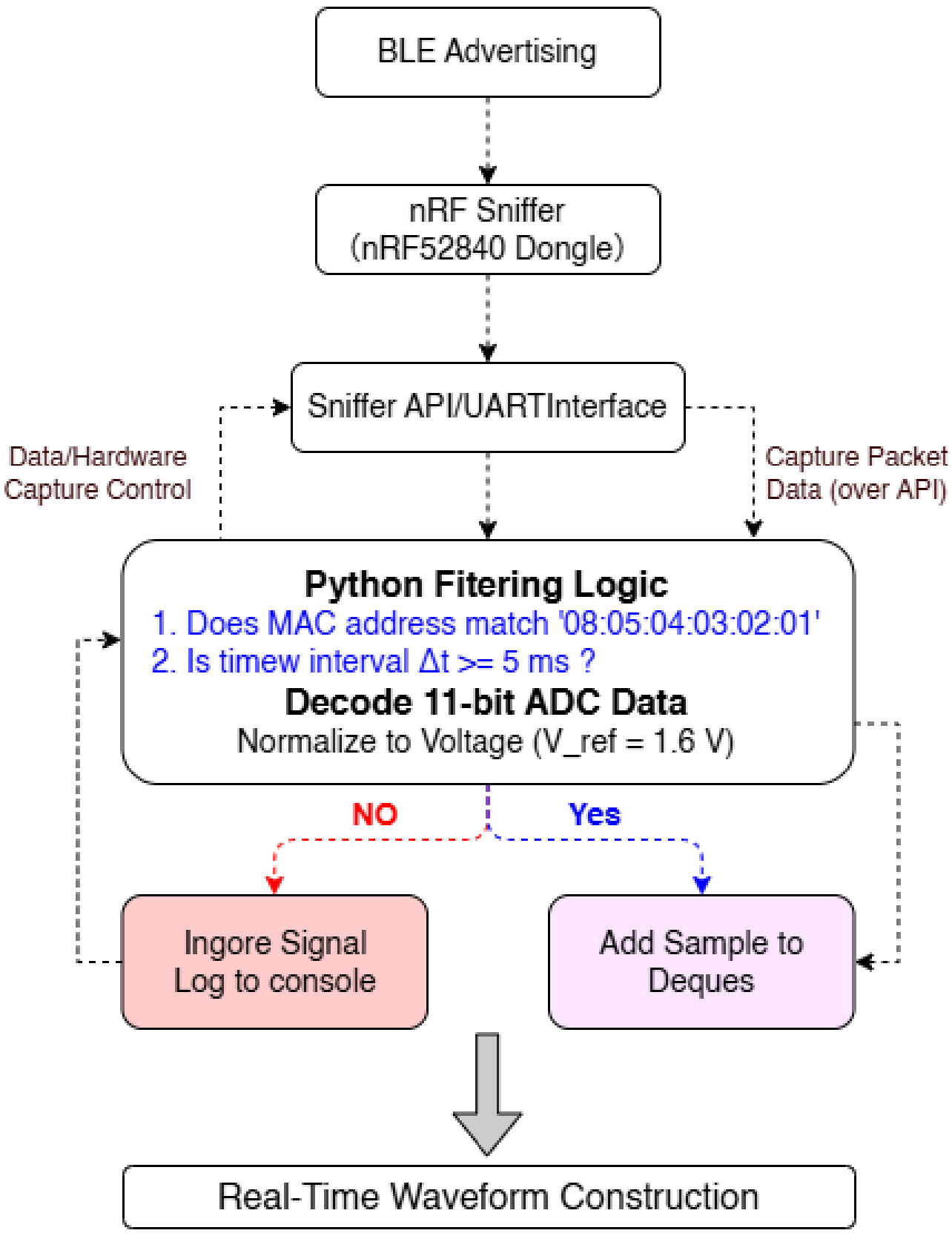

Figure S23. Flowchart illustrating the packet capture and data processing workflow at the receiving end.

Table S1. Detailed geometric and material properties of the components used in developing the self-powered sensing system.

Component	Parameter/Unit	Value
Spring steel	Width (mm)	22
	Length (mm)	170
	Thickness (mm)	0.8
	Material	65Mn
PZT-5A	Width (mm)	20
	Length (mm)	30
	Thickness (mm)	0.4
	Density (Kg/m^3)	7500
	Young's modulus (GPa)	56
	Capacitance (nF)	34.04
	Electromechanical coupling factors (NV)	3.16×10^{-5}
	Dielectric constants	3200
Mass	Diameter (mm)	44
	Length (mm)	60
	Thickness (mm)	3.5
	Weight (g)	33
	Material	Polylactic Acid (PLA)
PCB	Diameter (mm)	30
	Thickness (mm)	1 mm
	Weight (g)	18 g
Magnet	Diameter (mm)	12

	Thickness (mm)	5
	Material	NdFeB

Table S2. Comparison of continuous operation capability and effective sampling rates among representative self-powered IoT sensing nodes.

No.	Year	Harvester Type	Source	Vibration Frequency	Vibration Amplitude	Target Measurand	Operation Mode	Temporal Coverage	Effective Sampling Rate	Remark	Ref	Ref. in manuscript
1	2026	Piezoelectric	Shaker vibration	6-8 Hz	0.14 g	Vibration Waveforms	Continuous sensing	~ 100 %	~ 48 Sa/s	1 PZT, SECE circuits	This work	This work
2	2026	Piezoelectric	Shaker vibration	24 HZ	0.4 g	Temperature	Continuous sensing	100%	1 Sa/s	6 PZTs, SECE circuits	Adv. Energy Mater. (2026) 0:e06114	27
3	2026	Electromagnetic	Human motion (Arm and Leg)	N/A	N/A	Motion amplitude and angular	Duty-cycle sensing	N/A	N/A	4 EMGs (on leg/arm), ADC sampling:100 Hz	Device 4 (2026) 100972	34
4	2026	Hybrid (Electromagnetic& Triboelectric)	Magneto-mechanical energy	N/A	N/A	Environmental 5D sensing (Pressure, Temperature, Altitude, Humidity, and Lumination)	Intermittent sensing	N/A	~1.67 Sa/s	8 EMGs, 8 TENGs	Adv. Energy Mater. (2026), 16:e04837	25
5	2025	Hybrid (piezoelectric & Triboelectric)	Wind-induced vibrations	Galloping: ~5.4Hz; Flutter: 50Hz	N/A	Temperature	Intermittent sensing	N/A	~0.83 Sa/s	Wind speed = 10 m/s	Adv. Funct. Mater. (2025) 35, 2414324	19
6	2025	Piezoelectric	Shaker vibration	37 Hz	0.5 g	Acceleration, Temperature, and Voltage	Intermittent sensing	N/A	~0.2 Sa/s	12 Piezoelectric patches, SECE circuits	J. Sound Vib. (2025) 119609	29
7	2025	Piezoelectric	Wind-	7.85 Hz	N/A	Vibration waveform,	Duty-cycle	~1.3%	1.3 Sa/s	1 PZT,	Appl. Phys. Lett.	32

			induced vibrations			Wind speed	sensing			Wind speed = 4.486 m/s ADC sampling:100 Hz	(2025) 127, 203904	
8	2025	TENG	Wind-induced vibrations	N/A	N/A	Wind speed, Temperature, Humidity, and Noxious Gas (CO, NO_2, NH_4)	Intermittent sensing	N/A	0.016 Sa/s (wind speed)	10 arrays of Mixed-Mode TENGs Wind speed = 4.25 m/s, LoRa Communication	Nat Commun.(2025) 16(1): 5141	30
9	2025	TENG	Impact load	< 5Hz	N/A	Force monitoring	Continuous sensing	100%	1 MHz	Depend on external power supply	Sci. Adv. (2025) 11, eadw6179	38
10	2025	TENG	Wave	N/A	N/A	Temperature, PH	Intermittent sensing	N/A	0.67 Sa/s	wind speed:0.93 m/s; wave period: 1.93 s; wave height: 0.1 m,	Nat Commun.| (2025) 16:5480	51
10	2024	TENG	Wave	N/A	N/A	TDS value	Intermittent sensing	N/A	~0.003 Sa/s	in-situ ocean tests, LoRa communication	Nat Commun. (2024) 15:6834	24
11	2024	Electromagnetic	Wave	N/A	N/A	Temperature, PH, Water Level, Turbidity, conductivity,	Intermittent sensing	N/A	0.17 Sa/s	in-situ ocean tests wave frequency = 1-2 Hz	Nat Commun. (2024) 15:6513	44

12	2024	TENG	Ultrasound	20 kHz	N/A	Bluetooth Signal	Intermittent sensing	N/A	0.375 Sa/s	N/A	Joule (2024) 8(9): 2681-2695	49
13	2024	TENG	Diesel engine vibration	~ 45Hz		Frequency, Temperature, and Humidity	Intermittent sensing	N/A	~0.06 Sa/s	Lora communication	Adv. Energy Mater. (2024) 14, 2400585	48
14	2023	Piezoelectric	Shaker vibration	44 Hz	1 g	Temperature and Humidity	Intermittent sensing	N/A	0.043 Sa/s	1 PZT	ACS Nano (2023) 17, 6435−6451	50
15	2023	TENG	Wind	N/A	N/A	Temperature, Humidity, and Atmospheric Pressure	Duty-cycle sensing	N/A	~ 0.075 Sa/s	Under a moderate breeze<br>Each duty complete 1 to 3 times sensing	Adv. Energy Mater. (2023) 13, 2202691	47
16	2022	TENG	Wind	N/A	N/A	Temperature and Humidity	Intermittent sensing	N/A	0.033 Sa/s	Wind speed = 3 m/s	Adv. Funct. Mater. (2022) 32, 2205710	46
17	2021	TENG	Wind	~56.65 Hz	N/A	Wind Speed	Intermittent sensing	N/A	0.0083 Sa/s	Wind speed: 3 m/s	ACS Energy Lett. (2021), 6, 2343−2350	28
18	2021	TENG	Wave	N/A	N/A	Temperature and Humidity	Intermittent sensing	N/A	0.2 Sa/s	Wave frequency: 1.1 Hz<br>Wave amplitude: 10 cm	Adv. Funct. Mater (2021) 31, 2106398	45
19	2020	TENG	Human motion (Arm)	~1-2 Hz	N/A	pH and Na+ (Sweat)	Intermittent sensing	N/A	~0.005 Hz	9 km/h run on treadmill	Sci. Adv. (2020) 6: eaay9842	23

- Continuous sensing: Uninterrupted acquisition of consecutive samples throughout the observation period, without intentional sleep, recharge, or inactive gaps.
- Duty-cycle sensing: Periodic acquisition of consecutive data segments separated by predefined inactive or sleep intervals. A local waveform may be obtained during each active period, but the overall data stream contains temporal gaps.
- Intermittent sensing: Acquisition of isolated measurement points at separated time instants, typically after sufficient energy has accumulated. These discrete measurements do not form a continuous time-domain waveform.
- Temporal coverage: The proportion of the total observation period during which valid sensing data are acquired:

$$Temporal\,coverage = \frac{T_{active}}{T_{total}} \times 100\%$$

  where $\boldsymbol{T}_{\mathbf{active}}$ is the cumulative duration of valid data acquisition and $\boldsymbol{T}_{\mathbf{total}}$ is the total elapsed observation time, including inactive, sleep, and recharge intervals.
- Effective sampling rate: The average number of valid samples or measurement updates obtained per unit of total elapsed time:

$$f_{eff} = \frac{N_{valid}}{T_{total}}$$

  where N_{valid} is the total number of valid samples or measurement updates. For continuous sensing, the effective sampling rate is approximately equal to the nominal sampling rate. For duty-cycle and intermittent sensing, inactive or recharge intervals are included in T_{total}, and the effective sampling rate is therefore lower than the sampling rate used during an active period. When the available information is insufficient for a reliable calculation, the value is marked as “N/A.”

Supplementary Notes

Supplementary Note S1. Long-Term Cyclic Stability and Temperature-Dependent Robustness of the QZS Harvester.

1. Long-term cyclic stability

The magnetic negative-stiffness mechanism employed in the proposed QZS harvester is contactless and therefore does not cause frictional wear. The primary long-term reliability concerns are instead associated with the cyclic strain of the bonded PZT-5A patch, the integrity of the adhesive interface, fatigue of the spring-steel beam, and possible changes in the alignment or spacing of the magnetic assembly.

Representative technical data for PZT-5A reports a direct electromechanical coupling coefficient k_{33} of approximately 0.70-0.74, a planar coupling coefficient k_p of approximately 0.63-0.65, and a Curie temperature of approximately 270 °C [1]. These parameters provide the baseline material properties relevant to electromechanical conversion and thermal stability.

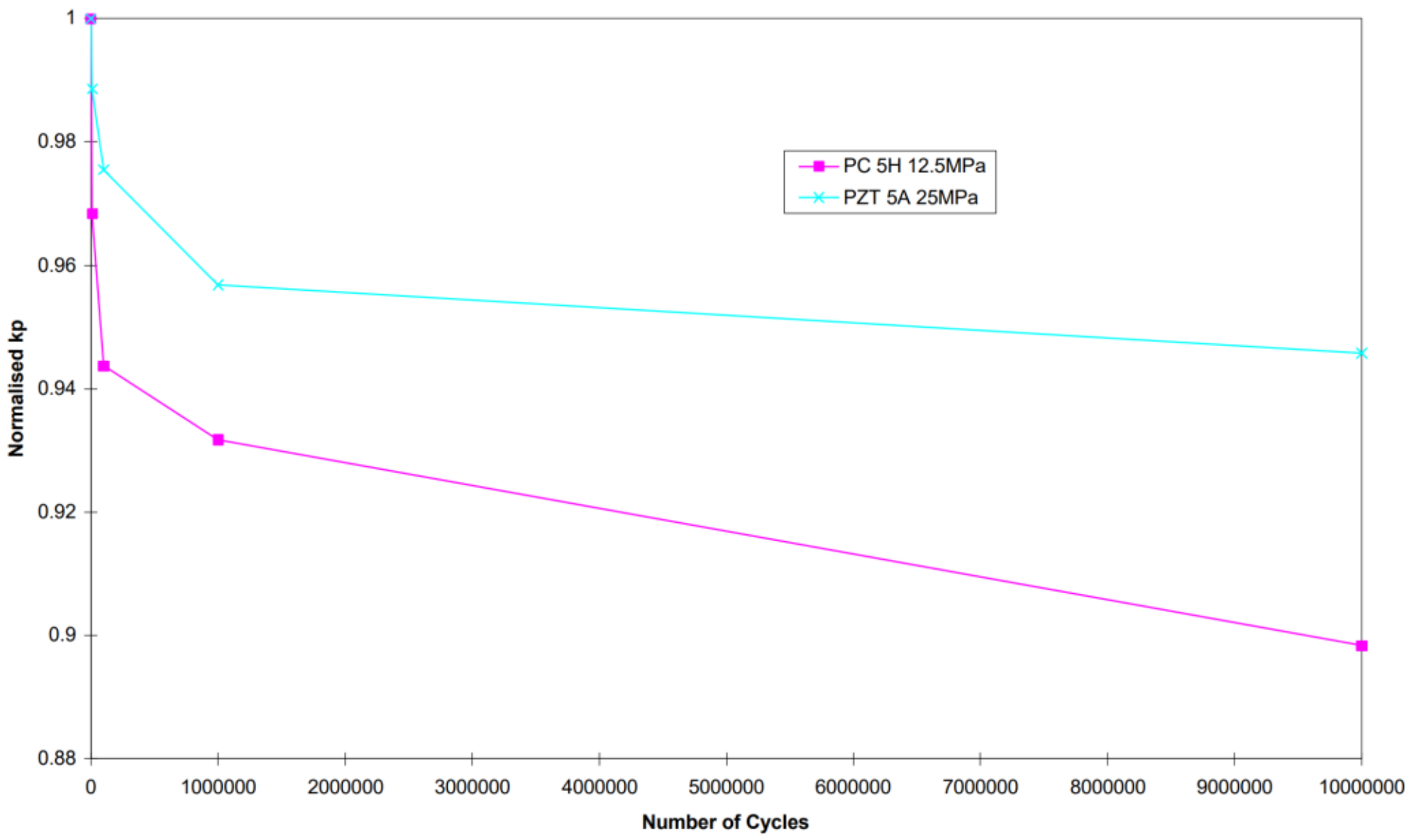

Figure R1. Representative mechanical-cycling behavior of PZT-5A reproduced from Figure 10 of Ref. [2]. Under a cyclic compressive stress of 25 MPa, the normalized planar electromechanical coupling coefficient, $\boldsymbol{k_p(N)/k_p(0)}$, of the reported PZT-5A specimens remained approximately 0.956 after 10^6 cycles and 0.945 after 10^7 cycles, corresponding to retention of approximately 95.6% and 94.5% of the initial value, respectively.

More importantly, mechanical-cycling tests reported by the National Physical Laboratory indicate that the degradation of PZT-5A depends strongly on the applied cyclic stress and specimen geometry, rather than on cycle number alone [2]. For PZT-5A discs with a diameter of 10 mm and a thickness

of 1 mm subjected to a cyclic compressive stress of 25 MPa, the normalized planar electromechanical coupling coefficient k_p remained approximately 0.956 after 10^6 cycles and approximately 0.945 after 10^7 cycles. In other words, approximately 95.6% and 94.5% of the initial coupling performance were retained after one million and ten million cycles, respectively [2]. The reported degradation became substantially more pronounced at higher cyclic stress levels, indicating that controlling the local mechanical stress is more meaningful for lifetime prediction than specifying a universal cycle-life value.

These results indicate that PZT-5A can retain a high level of electromechanical coupling performance over millions of cycles when the applied stress is appropriately controlled. In the proposed harvester, the PZT patch operates through bending-induced strain in a bonded PZT–steel structure. Therefore, limiting local strain and stress concentration while maintaining the integrity of the adhesive interface and mechanical fixation is important for long-term operation.

2. Temperature-induced magnetic drift

The QZS condition is established by balancing the positive stiffness of the cantilever beam with the negative stiffness generated by the repulsive N52 magnets. Temperature-induced changes in the magnetic properties may therefore disturb this stiffness balance and alter the location and width of the QZS region.

Standard Characteristics

Items			Materials	N52 C// (C⊥)
Magnetic Properties	Remanence B_r		[T]	1.40 ~ 1.47
			[G]	14000 ~ 14700
	Coercivity	H_{cB}	[kA / m]	820 ~
			[Oe]	10300 ~
		H_{cJ}	[kA / m]	875 ~
			[Oe]	11000 ~
	Maximum Energy Product $(BH)_{max}$		[kJ / m^3]	378 ~ 422
			[MGOe]	47.5 ~ 53
	Recoil Permeability μ_r		[-]	1.05
Temperature Properties	Thermal Coefficient of B_r *1		[% / K]	- 0.11
	Thermal Coefficient of H_{cJ} *2		[% / K]	- 0.61
	Coefficient of Thermal Expansion *3		[1 / K]	6.7×10^{-6} (-2.8×10^{-6})
	Curie Point		[°C]	330

Figure R2. Manufacturer-specified magnetic and thermal properties of N52 NdFeB magnets. The reversible temperature coefficient of remanence is approximately -0.11%/K, while the temperature coefficient of intrinsic coercivity is approximately -0.61%/K. Cropped from the “Standard Characteristics” table on page 2 of Ref. [3].

According to the manufacturer-provided N52 material data, the remanence B_r is approximately 1.40-1.47 T, the reversible temperature coefficient of remanence is approximately $-0.11\%/\text{K}$, and the temperature coefficient of intrinsic coercivity is approximately $-0.61\%/\text{K}$ [3]. The reported Curie temperature is approximately 350 °C [3].

Based on the reported reversible temperature coefficient, an increase in temperature from 20 to 60 °C would correspond to an estimated reduction in remanence of

$$\frac{\Delta B_r}{B_r} \approx -0.11\% \times (60-20) = -4.4\%$$

This estimated variation is reversible within the specified operating range. The exact variation in the magnetic interaction and negative stiffness of the QZS assembly additionally depends on the magnet geometry, spacing, alignment, working point, and thermal expansion of the supporting structure.

A reduction in remanence weakens the repulsive magnetic interaction and decreases the magnitude of the negative stiffness provided by the magnets. Consequently, the compensation of the beam's positive stiffness becomes less complete, potentially increasing the equivalent stiffness, shifting or narrowing the QZS region, and moving the high-energy response away from the originally designed frequency range. The resulting reduction in vibration amplitude would decrease the usable harvested-power margin.

Conversely, decreasing the temperature within the reversible range increases the magnet remanence and strengthens the magnetic interaction. However, an excessive increase in negative stiffness may also disturb the designed stiffness balance. Therefore, both positive and negative temperature deviations can shift the optimal QZS operating condition.

3. Implications for the system power budget

The influence of cyclic degradation and temperature-induced magnetic drift on the complete sensing system is ultimately determined by the remaining power margin. A reduction in the piezoelectric coupling performance would decrease the mechanical-to-electrical conversion capability, whereas a temperature-induced change in magnetic negative stiffness could shift the QZS response away from the excitation frequency. Both effects may reduce the usable harvested power.

Under the demonstrated operating condition, the continued increase in V_{store} after node activation in Figure 5D indicates that the system retains a positive power margin. If the degradation- or temperature-induced reduction in harvested power remains smaller than this margin, sustained sensing can still be maintained. However, if the usable harvested power falls below the total

consumption of sensing, data processing, wireless transmission, and residual circuit losses, V_{store} will gradually decrease. Once it falls below the falling UVLO threshold, the system will transition from sustained operation to intermittent recharge–restart operation.

For practical engineering applications involving long service durations or wider temperature ranges, possible mitigation strategies include limiting local PZT strain and stress concentration, improving the durability of the adhesive interface and magnetic fixation, employing thermally stabilized or higher-coercivity magnets, and introducing adjustable magnet spacing to compensate for variations in magnetic negative stiffness.

4. Limitations and applicability

The reported PZT-5A cycling data and N52 temperature coefficients provide quantitative support for evaluating the expected long-term and thermal behavior of the proposed QZS harvester. Nevertheless, the durability of the integrated device is also influenced by the local bending strain of the bonded PZT patch, fatigue of the spring-steel beam, long-term integrity of the adhesive interface, and possible changes in the alignment or spacing of the magnetic assembly.

Similarly, the estimated temperature-induced change in remanence indicates the expected variation in magnetic negative stiffness, whereas the exact shift of the QZS region also depends on the magnet geometry, spacing, mechanical tolerances, and the thermal expansion of the supporting structure. Therefore, application-specific endurance and environmental qualification of the integrated harvester will remain necessary for deployment under long service durations, large temperature variations, or highly variable vibration conditions.

References related to this Note:

[1] PIEZO Systems, Inc. PZT5A & 5H Materials Technical Data (Typical Values). PIEZO Systems, Inc.

[2] Cain, M. G.; Stewart, M.; Gee, M. G. Degradation of Piezoelectric Materials. NPL Report CMMT(A) 148, National Physical Laboratory, Teddington, UK, 1999.

[3] Shin-Etsu Chemical Co., Ltd. Nd–Fe–B Magnet N52: Standard Characteristics and Demagnetization Curves at Elevated Temperature. Shin-Etsu Chemical Co., Ltd., 2018.
